# KLF family members control expression of genes required for tissue macrophage identities

**DOI:** 10.1084/jem.20240379

**Published:** 2025-03-12

**Authors:** Kathleen Pestal, Leianna C. Slayden, Gregory M. Barton

**Affiliations:** 1 Division of Immunology and Molecular Medicine, Department of Molecular and Cell Biology, University of California, Berkeley, CA, USA; 2 Howard Hughes Medical Institute, University of California, Berkeley, Berkeley, CA, USA

## Abstract

Tissue-resident macrophages adopt distinct gene expression profiles and exhibit functional specialization based on their tissue of residence. Recent studies have begun to define the signals and transcription factors that induce these identities. Here we describe an unexpected and specific role for the broadly expressed transcription factor Krüppel-like factor 2 (KLF2) in the development of embryonically derived large cavity macrophages (LCMs) in the serous cavities. KLF2 not only directly regulates the transcription of genes previously shown to specify LCM identity, such as retinoic acid receptors and GATA6, but also is required for induction of many other transcripts that define the identity of these cells. Our results suggest that KLF4 may similarly regulate the identity of alveolar macrophages in the lung. These data demonstrate that broadly expressed transcription factors, such as group 2 KLFs, can play important roles in the specification of distinct identities of tissue-resident macrophages.

## Teaser

This work demonstrates that group 2 KLF family transcription factors are critical for specifying the identity of distinct tissue-resident macrophages. KLF2 directly controls expression of genes previously shown to be necessary in cavity macrophages, while KLF4 may play a similar role in alveolar macrophages.

## Introduction

Tissue-resident macrophages develop from yolk sac progenitors during embryogenesis (Gomez Perdiguero et al., 2015; Mass et al., 2016; Stremmel et al., 2018). The essential macrophage growth factor M-CSF (CSF-1) induces these progenitors to differentiate into early macrophages that seed the developing tissues where they encounter additional, tissue-specific signals that drive the expression of distinct tissue phenotypes and functions (Gautier et al., 2012; Gosselin et al., 2014; Lavin et al., 2014). Previous studies have demonstrated the importance of such tissue-specific signals by performing macrophage transfers between tissues or tracking differentiation of monocytes as they repopulate a tissue after perturbation (Lavin et al., 2014; Roberts et al., 2017; Sakai et al., 2019). For some tissue-resident macrophage populations, key transcription factors have been identified that respond to tissue-specific signals and control enhancer activity and gene expression, ultimately leading to the discrete functional characteristics of each population (Buttgereit et al., 2016; Guilliams et al., 2013; Okabe and Medzhitov, 2014; Sakai et al., 2019; Schneider et al., 2014). However, for most tissue-resident macrophage populations, our understanding of the transcriptional networks required for this specification remains incomplete.

The Krüppel-like factors (KLFs) are a family of 17 broadly expressed eukaryotic zinc finger transcription factors consisting of three subgroups (Pollak et al., 2018; Yuce and Ozkan, 2024). The KLFs are involved in many different aspects of biology, functioning as both repressors and activators of transcription. In several studies cataloging the different molecular identities of tissue-resident macrophages, an enrichment in KLF transcription factor–binding sites was noted in regions of active and open chromatin of myeloid cell subsets, including circulating monocytes, macrophages, and neutrophils (Lavin et al., 2014). KLF-binding sites were enriched in genomic regions bound by the myeloid pioneering factor PU.1 in large cavity macrophages (LCMs) (Gosselin et al., 2014), suggesting that KLFs could be involved during the development of these macrophages; however, these studies did not address the function of KLFs. In fact, previous work examining the function of KLFs in macrophages concluded that KLF2 and KLF4 repress the activation and polarization of macrophages (Das et al., 2006, 2012; Liao et al., 2011; Mahabeleshwar et al., 2011; Roberts et al., 2017; Shi et al., 2014; Sweet et al., 2020). Therefore, any contribution that KLFs play in early macrophage development and specification remains unexplored.

Here we focus primarily on macrophage populations from the serous cavities and the lung. The appropriate induction of LCMs in the peritoneal, pleural, and pericardial serous cavities has previously been shown to require retinoic acid signaling and the expression of the transcription factor GATA6 (Buechler et al., 2019; Casanova-Acebes et al., 2020; Gautier et al., 2012, 2014; Gosselin et al., 2014; Okabe and Medzhitov, 2014; Rosas et al., 2014). Development of alveolar macrophages (AMs) in the lung is the result of early macrophages receiving GM-CSF (CSF-2) and TGF-β signals as well as the induction of the transcription factors C/EBPβ and PPARγ (Dorr et al., 2022; Schneider et al., 2014; Yu et al., 2017). Our group previously identified a role for two group 2 KLFs, KLF2 and KLF4, in dampening innate immune responses against nucleic acids within apoptotic cell corpses engulfed by LCMs (Roberts et al., 2017), which motivated us to explore a role for these transcription factors more broadly in the function of tissue macrophages.

In this study, we show that KLF2 is critically important for the development of LCMs, and we provide evidence that KLF4 may play a similar role in AMs. KLF2 deficiency results in a nearly complete absence of LCMs in vivo due to an inability to recognize signals in the cavity environment, a phenotype distinct from what is observed in GATA6-deficient LCMs. We demonstrate that KLF2 directly regulates a number of LCM genes, including genes previously implicated in specifying LCM identity, such as GATA6 and retinoic acid receptors. Similarly, deficiency in KLF4 impaired development of AMs, and the remaining cells failed to express many known AM genes. Collectively, these results establish the importance of group 2 KLFs for the induction of tissue-resident macrophage identities.

## Results

### KLF2 is required for the development of serous LCMs

The serous cavities contain two populations of macrophages commonly referred to as small cavity macrophages (SCMs; entirely monocyte derived) and LCMs (embryonic derived, “tissue-resident” macrophages). These two populations are phenotypically distinct and easily identified by surface markers (Bain and Jenkins, 2018; Ghosn et al., 2010; Kim et al., 2016; Nguyen et al., 2012). SCMs are CD11b^+^F4/80^mid/lo^MHCII^hi^DNAM-1^hi^ICAM2^−^ and LCMs are CD11b^+^F4/80^hi^MHCII^−^DNAM-1^−^ICAM2^+^. Previous studies have shown a specific expression of GATA6 in LCMs relative to other tissue-resident macrophage populations (Gosselin et al., 2014; Lavin et al., 2014; Okabe and Medzhitov, 2014). Locally produced retinoic acid is required for GATA6 expression, and lack of GATA6 leads to a change in phenotype, function, and the transcriptional landscape of LCMs (Buechler et al., 2019; Casanova-Acebes et al., 2020; Okabe and Medzhitov, 2014). Based on the high expression of KLF2 in LCMs and our previous work suggesting KLF2 is functionally relevant in these cells (Roberts et al., 2017), we investigated the relative importance of KLF2 versus GATA6 in LCMs. We analyzed immune cells isolated from the peritoneal and pleural serous cavities of mice lacking KLF2 or GATA6 in myeloid cells by crossing *Klf2*-floxed mice or *Gata6*-floxed mice to LysMCre mice (*LysM*^*Cre/+*^*Klf2*^*fl/fl*^, and *LysM*^*Cre/+*^*Gata6*^*fl/fl*^).

**Figure 1.**
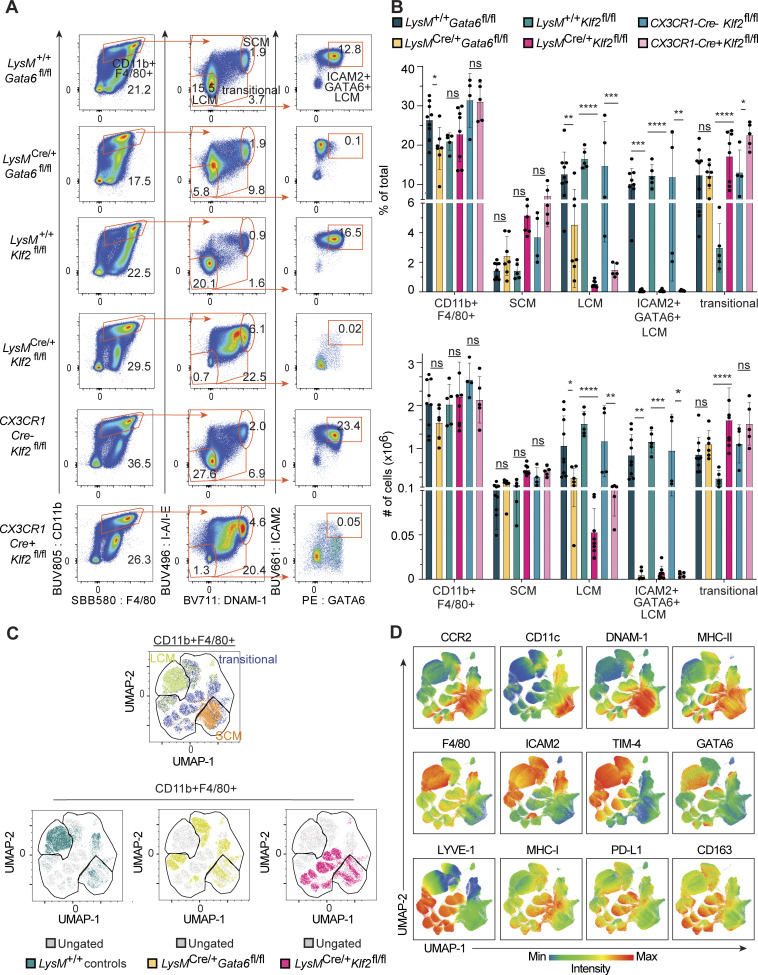
**Impaired development of LCMs in myeloid-specific KLF2-deficient mice. (A)** Flow cytometry of cells in peritoneal cavity lavage from the indicated mice, gated on live, single, CD3^−^B220^−^CD19^−^ cells. Numbers adjacent to gates indicate the percentage of total live events for each gate. **(B)** Bar graphs depicting the percentage of total live cells and total number of cells for the following populations (representative gates shown in A) as measured by flow cytometry: CD11b^+^F4/80^+^, SCM (CD11b^+^F4/80^+^MHCII^hi^DNAM-1^hi^), LCMs (CD11b^+^F4/80^+^MHCII^−^DNAM-1^−^), ICAM2^+^GATA6^+^ LCM (CD11b^+^F4/80^+^MHCII^−^DNAM-1^−^ICAM2^+^GATA6^+^), and transitional (CD11b^+^F4/80^+^MHCII^mid^DNAM-1^mid^). **(C)** Dimensionality reduction via UMAP of combined CD11b^+^F4/80^+^ cells from *LysM*^*+/+*^*Gata6*^*fl/fl*^ and *LysM*^*+/+*^*Klf2*^*fl/fl*^ (gated together as *LysM*^*+/+*^ controls), *LysM*^*Cre/+*^*Gata6*^*fl/fl*^, or *LysM*^*Cre/+*^*Klf2*^*fl/fl*^ mice, downsampled to normalize cells per genotype. The resulting projection was overlayed with supervised gates for LCMs, transitional, and SCMs as shown in A. The upper panel shows all cells, while the lower panels show only cells from the indicated genotypes. **(D)** Heatmaps of defining marker expression for SCM, LCM, and transitional cell clusters overlaid onto the UMAP projections from C. Data in A are from one experiment representative of six independent experiments; data in B are combined from six independent experiments, *LysM*^+/+^*Gata6*^*fl/fl*^ (*n* = 9), *LysM*^*Cre/+*^*Gata6*^*fl/fl*^ (*n* = 6), *LysM*^+/+^*Klf2*^*fl/fl*^ (*n* = 5), *LysM*^*Cre/+*^*Klf2*^*fl/fl*^ (*n* = 7), CX3CR1-Cre^−^*Klf2*^*fl/fl*^ (*n* = 4), and CX3CR1-Cre^+^*Klf2*^*fl/fl*^ (*n* = 5). Significance determined by ordinary two-way ANOVA with multiple comparisons and Šidák’s correction. Asterisks denote: ****P < 0.0001, ***P = 0.0006, **P = 0.0021, and *P = 0.033.

**Figure S1.**
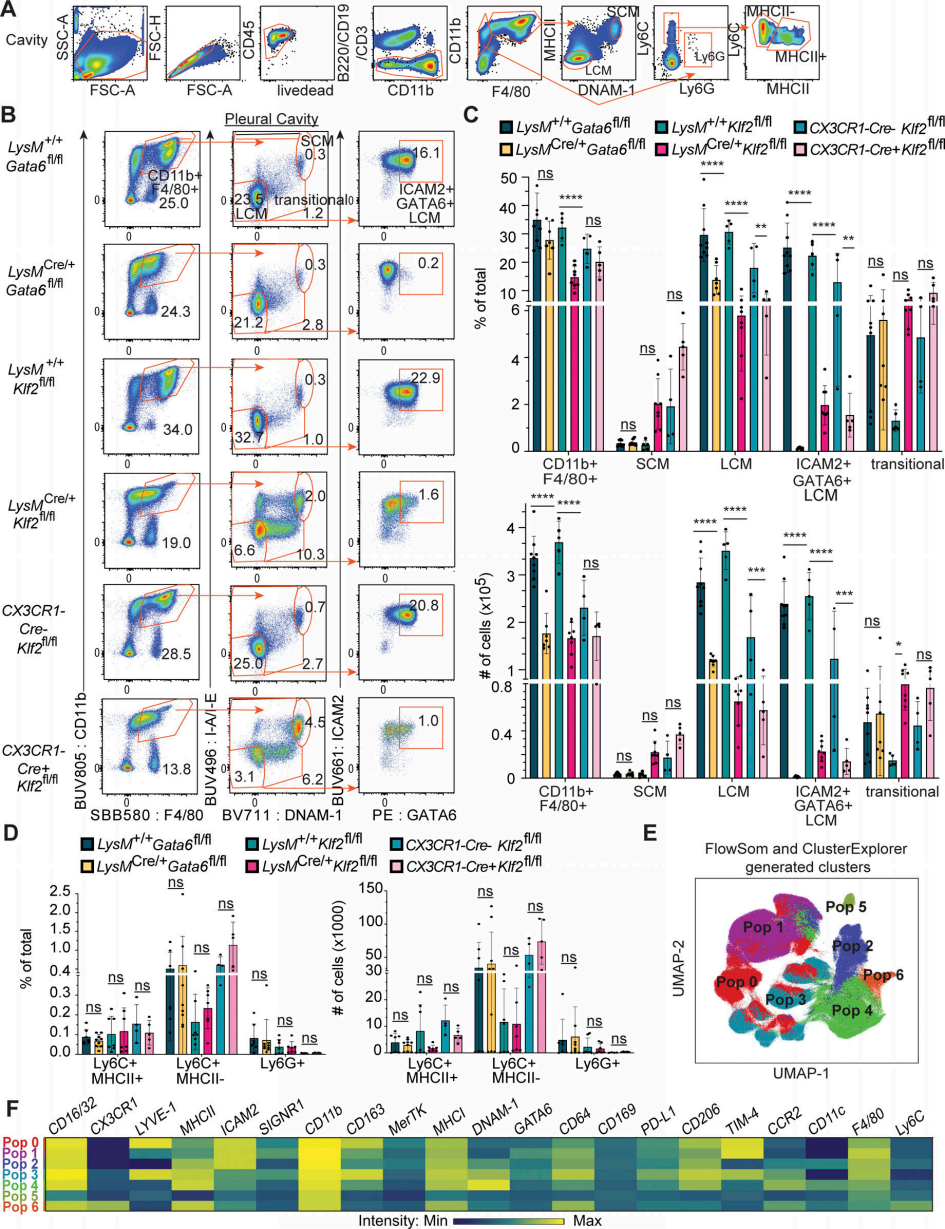
**Effects of KLF2 deficiency on myeloid cells in the pleural and peritoneal cavities. (A)** Flow cytometry gating strategy for cavities. **(B)** Flow cytometry of cells in pleural cavity lavage from the indicated mice, gated on live, single, CD3^−^B220^−^CD19^−^ cells. Numbers adjacent to gates indicate the percentage of total live events for each gate. **(C)** Bar graphs depicting the percentage of total live cells and total number of cells for the following populations in the pleural cavities (representative gates shown in A) as measured by flow cytometry: CD11b^+^F4/80^+^, SCM (CD11b^+^F4/80^+^MHCII^hi^DNAM-1^hi^), LCMs (CD11b^+^F4/80^+^MHCII^−^DNAM-1^−^), ICAM2^+^GATA6^+^ LCMs (CD11b^+^F4/80^+^MHCII^−^DNAM-1^−^ICAM2^+^GATA6^+^), and transitional (CD11b^+^F4/80^+^MHCII^mid^DNAM-1^mid^). **(D)** Bar graphs depicting the percent of total and total number of monocytes (CD11b^+^F4/80^−^Ly6C^+^MHCII^+^ or MHCII^−^) and neutrophils (CD11b^+^F4/80^−^, Ly6G^+^), as measured by flow cytometry. **(E and F)** FlowSOM and ClusterExplorer-derived unsupervised cluster identification (E) and heatmap of marker intensities defining identified clusters (F). Significance determined by ordinary two-way ANOVA with multiple comparisons and Šidák’s correction. Asterisks denote: ****P < 0.0001, ***P = 0.0006, **P = 0.0021, and *P = 0.033.

In unmanipulated adult WT B6 mice, most cavity macrophages (>80% of CD11b^+^F4/80^+^ cells) are LCMs and <10% are SCMs ([Bain and Jenkins, 2018] and Fig. 1 A). As previously described, GATA6 deficiency reduced the total percentage of macrophages (CD11b^+^F4/80^+^) in the peritoneal and pleural serous cavities; this reduction was mostly due to a lower frequency and number of LCMs (Fig. 1, A and B; and Fig. S1, A–C). The remaining LCMs expressed lower levels of F4/80, but otherwise expressed surface markers characteristic of WT LCMs despite expressing no GATA6 (Fig. 1 A and Fig. S1 B). Thus, while reduced in number, the remaining *Gata6*-deficient cells largely resemble LCMs, as previously described (Gautier et al., 2014; Okabe and Medzhitov, 2014; Rosas et al., 2014). *LysM*^*Cre/+*^*Gata6*^*fl/fl*^ mice showed no significant changes to the frequency or number of SCMs and a population of transitional cells with intermediate levels of DNAM-1 and MHCII (DNAM1^mid^MHCII^mid^) (Fig. 1, A and B). These transitional cells have previously been described as “naïve converting cavity cells” to distinguish them from “converting cavity cells” that accumulate in the cavity during infection (Finlay et al., 2023). In recognition of their relevance during homeostasis, we instead refer to them as “transitional cells” here. Additionally, the percentages and numbers of cavity monocytes (CD11b^+^Ly6C^+^MHC^+/−^) and neutrophils (CD11b^+^Ly6G^+^) were unchanged relative to WT controls (Fig. S1 D).

In contrast, *LysM*^*Cre/+*^*Klf2*^*fl/fl*^ mice exhibited a severe reduction in LCMs and a corresponding increase in transitional cells (Fig. 1, A and B). The very few remaining CD11b^+^F4/80^+^MHCII^−^DNAM-1^−^ cells (representing <1% of total cells in the cavity relative to ∼17% in control mice) did not express ICAM2 or GATA6 and had lower levels of F4/80, suggesting that they are not bona fide LCMs and that no such cells exist in the serous cavities of *LysM*^*Cre/+*^*Klf2*^*fl/fl*^ mice (Fig. 1, A and B; and Fig. S1, A–C). The frequency and absolute number of monocytes and neutrophils (CD11b^+^Ly6C^+^MHCII^+/−^) were unchanged in *Klf2*-deficient mice, showing that KLF2 is not required for the development of either cell type (Fig. S1 D).

While LysMCre is commonly used to delete genes efficiently in serous cavity macrophages, we independently validated the importance of KLF2 in these cells by crossing *Klf2*^*fl/fl*^ mice to *CX3CR1Cre* mice. Similar to *LysM*^*Cre/+*^*Klf2*^*fl/fl*^ mice, the frequency and number of LCMs in *CX3CR1Cre*^*+*^*Klf2*^*fl/fl*^ mice was dramatically reduced compared with *CX3CR1Cre*^*−*^*Klf2*^*fl/fl*^ control mice, and the few remaining cells lacked expression of both GATA6 and ICAM2 (Fig. 1, A and B; and Fig. S1, B and C).

To further characterize the macrophages in the serous cavities, we performed dimensionality reduction analysis of high-parameter flow cytometry data on equal numbers of total CD11b^+^F4/80^+^ macrophages from *LysM*^*+/+*^*Gata6*^*fl/fl*^, *LysM*^*+/+*^*Klf2*^*fl/fl*^, *LysM*^*Cre/+*^*Gata6*^*fl/fl*^, and *LysM*^*Cre/+*^*Klf2*^*fl/fl*^ mice via uniform manifold approximation and projection (UMAP). Layering our supervised LCMs, SCMs, and transitional cell gates onto the UMAP identified which clusters represent the cells from those gates (Fig. 1 C). We also applied the FlowSOM algorithm to the concatenated CD11b^+^F4/80^+^ cells to generate unsupervised identification of populations within our data, which we visualized with ClusterExplorer in FlowJo. FlowSOM identified seven populations (Fig. S1 E) of cavity macrophages based on the expression of 21 markers (Fig. S1 F). Because these populations align well with our supervised gates, this analysis confirmed our more general supervised identification of three major populations of cavity macrophages.

The cells from *LysM*^*+/+*^*Gata6*^*fl/fl*^ and *LysM*^*+/+*^*Klf2*^*fl/fl*^ mice cluster together (Fig. 1, C and D), with the majority of macrophages clustering as LCMs (F4/80^hi^ICAM2^+^TIM-4^+^GATA6^+^) and a small number of cells falling into the SCMs (CCR2^+^CD11c^+^DNAM-1^+^MHCII^+^) and transitional cell (LYVE-1^+^MHCI^hi^PDL-1^hi^CD163^hi^) clusters, as expected for WT mice. The majority of *LysM*^*Cre/+*^*Gata6*^*fl/fl*^ cells fall into clusters directly adjacent to the LCMs cluster. These clusters are characterized by lower expression of F4/80 and no expression of GATA6 but maintain expression of ICAM2 and TIM-4 (Fig. 1, C and D). In contrast, the *Klf2*-deficient CD11b^+^F4/80^+^ cells cluster distinctly from both the WT and *Gata6*-deficient cells. Almost no cells fall into the LCM or LCM adjacent clusters and instead cluster nearly exclusively in the transitional and SCM populations (Fig. 1, C and D). Altogether, these data further support the finding that *Klf2*-deficient macrophages in the serous cavities are incapable of developing into LCMs and instead remain in a transitional state that is mostly absent in WT and *Gata6*-deficient cells.

### The requirement for KLF2 in LCMs is cell intrinsic

**Figure 2.**
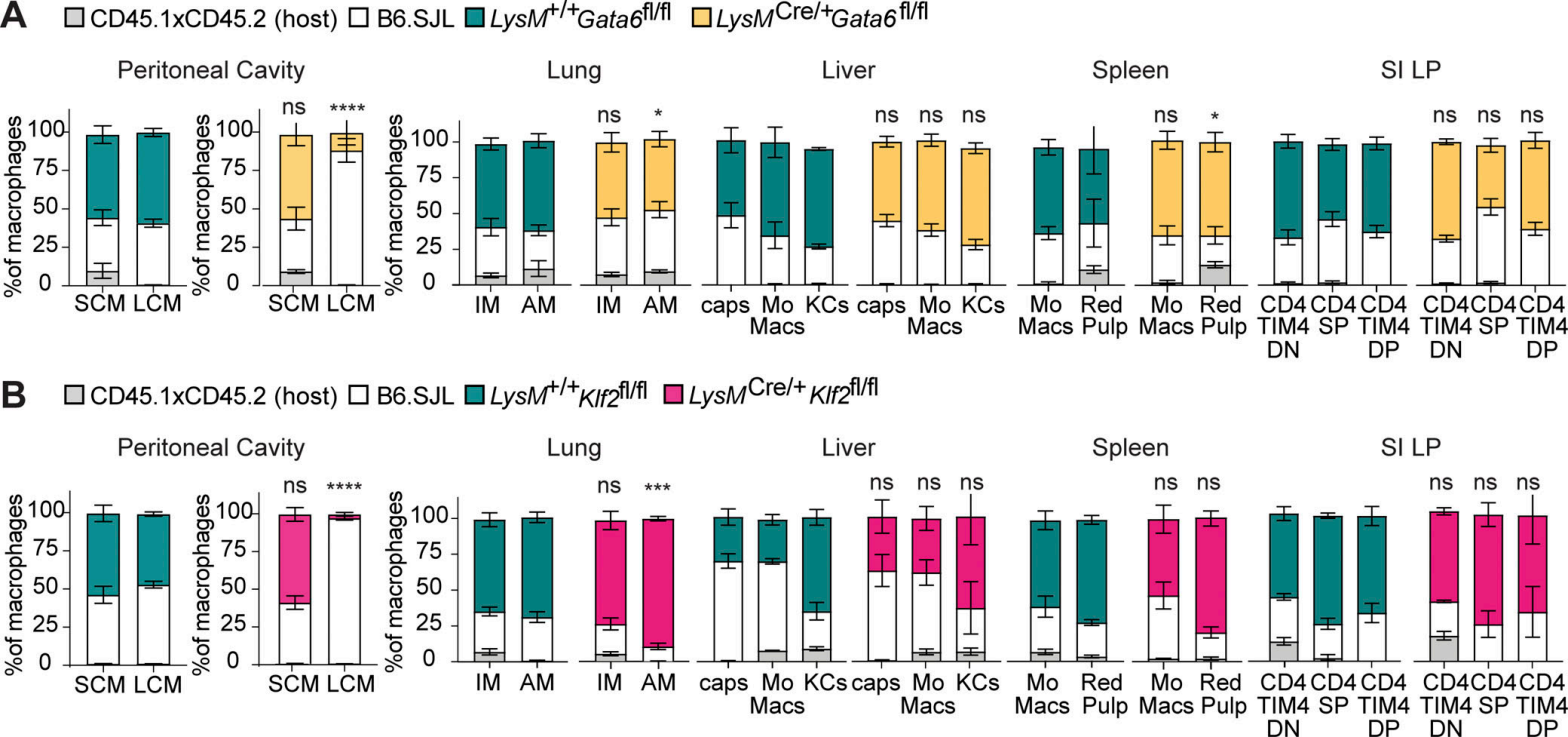
**Inability of KLF2-deficient progenitors to develop into LCMs is cell intrinsic. (A)** Frequency of cells of the indicated genotypes within SCM, LCM, and monocyte and resident macrophage subsets in the indicated tissues from radiation chimeras generated with *LysM*^*+/+*^*Gata6*^*fl/fl*^:B6.SJL or *LysM*^*Cre/+*^*Gata6*^*fl/fl*^:B6.SJL mixtures of donor bone marrow into CD45.1 × CD45.2 hosts. **(B)** Frequency of cells of the indicated genotypes within SCM, LCM, and monocyte and resident macrophage subsets in the indicated tissues from radiation chimeras generated with *LysM*^*+/+*^*Klf2*^*fl/fl*^:B6.SJL or *LysM*^*Cre/+*^*Klf2*^*fl/fl*^:B6.SJL mixtures of donor bone marrow into CD45.1 × CD45.2 hosts. Data in A and B are from one experiment representative of two independent experiments and presented as mean with SD of three to four chimeric animals per group. Significance determined by Fisher’s exact test of relative contributions of each genotype in B6.SJL:*LysM*^*Cre/+*^ chimeras compared with B6.SJL:*LysM*^*+/+*^ chimeras. Asterisks denote: ****P < 0.0001, ***P = 0.0006, and *P = 0.033.

**Figure S2.**
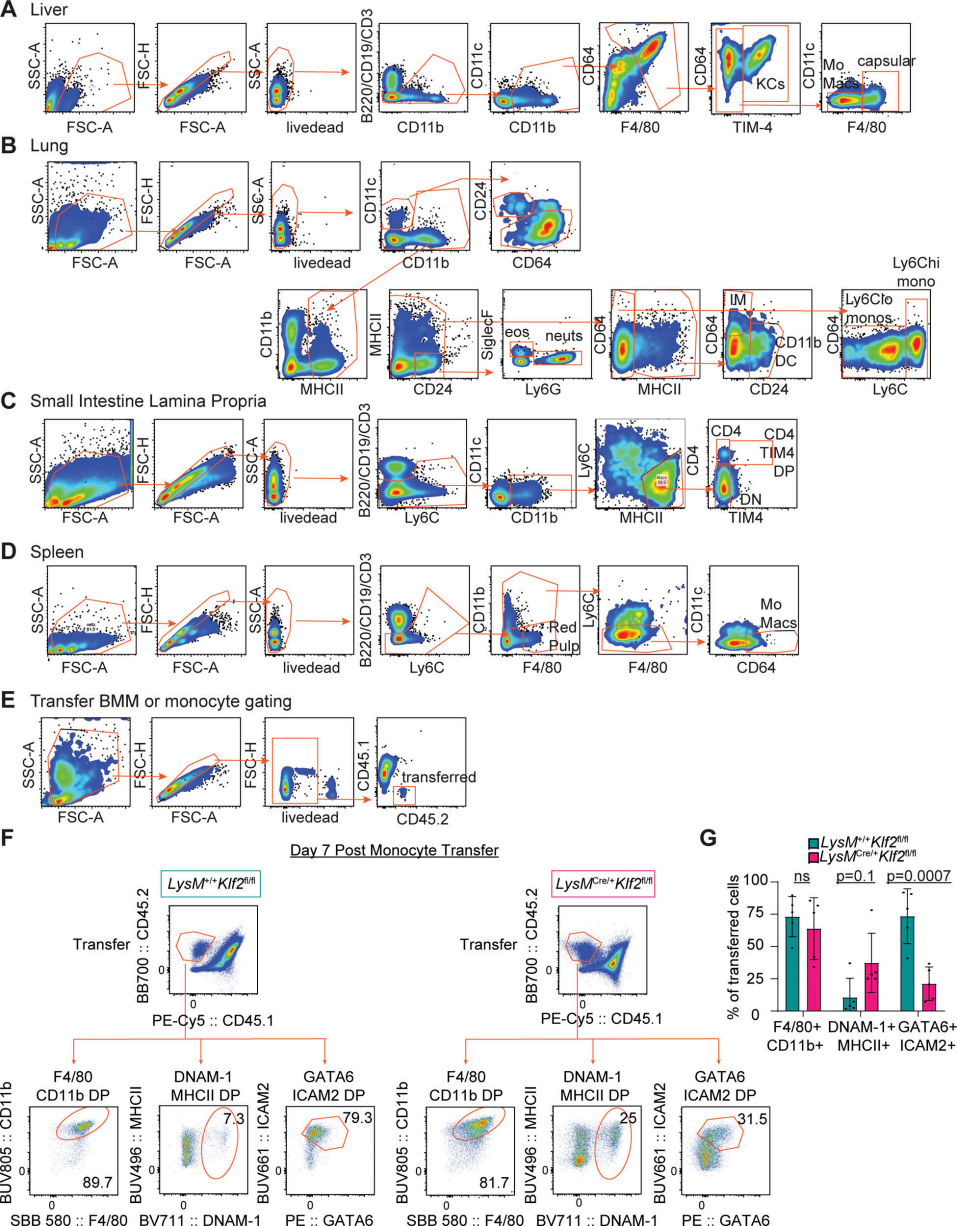
**Identification of tissue myeloid populations and transferred cell phenotypes. (A–D)** Flow cytometry gating for (A) liver, (B) lung, (C) small intestine lamina propria, and (D) spleen. **(E)** Flow cytometry gating for transferred cell experiments. **(F)** Representative flow cytometry analysis showing marker expression on purified bone marrow monocytes from *LysM*^*+/+*^*Klf2*^*fl/fl*^ or *LysM*^*Cre/+*^*Klf2*^*fl/fl*^ mice 7 days after transfer into a precleared peritoneal cavity of congenitally marked (CD45.1 × CD45.2) mice. **(G)** Bar graph depicting the percentage of total transferred cells of gates from F. Data from three combined experiments of transferred bone marrow monocytes into peritoneal cavities. Significance determined by ordinary two-way ANOVA with multiple comparisons and Šidák’s correction.

We next examined the requirement for KLF2 in LCMs when competing with WT cells by making radiation bone marrow chimeras using mixed *LysM*^*+/+*^*Klf2*^*fl/fl*^ or *LysM*^*Cre/+*^*Klf2*^*fl/fl*^ donor bone marrow. Again, we included *Gata6*-deficient cells as a comparison, as well as control radiation chimeras with mixtures of congenitally distinct control bone marrow. Both *Klf2*-deficient and *Gata6*-deficient cells failed to compete with WT cells in the generation of LCMs, while SCMs were equally comprised of WT and deficient cells (Fig. 2, A and B). The reduction of LCMs was more complete for *Klf2*-deficient cells than *Gata6*-deficient cells (Fig. 2, A and B); however, the difference between the genotypes was minor when compared with our analysis of *LysM*^*Cre/+*^*Klf2*^*fl/fl*^ and *LysM*^*Cre/+*^*Gata6*^*fl/fl*^ mice at homeostasis (Fig. 1). In control mice that received a mixture of bone marrow from two WT mice, both the SCM and LCM populations had equal contributions from each donor. Importantly, loss of either GATA6 or KLF2 had no significant effect on the development of any other tissue-resident macrophage populations in the liver, spleen, lung, and small intestine (Fig. 2, A and B; and Fig. S2, A–D).

These results, together with the analyses in Fig. 1, suggest an essential, cell-intrinsic role for KLF2 in the development of LCMs that appears to be distinct from the role played by GATA6. As an independent measure of the cell-intrinsic requirement for KLF2, we transferred purified monocytes from *LysM*^*+/+*^*Klf2*^*fl/fl*^ or *LysM*^*Cre/+*^*Klf2*^*fl/fl*^ mice into precleared peritoneal cavities of congenitally distinct recipient mice and harvested peritoneal cells 7 days later to evaluate the developmental potential of the transferred cells. Most of the transferred *LysM*^*+/+*^*Klf2*^*fl/fl*^ cells upregulated GATA6 and ICAM2, consistent with development into LCMs. In contrast, the recovered *Klf*2-deficient cells failed to upregulate these LCM markers, and a higher percentage of the macrophages expressed both MHCII and DNAM-1, consistent with the cells failing to develop beyond SCMs and/or transitional cells (Fig. S2, E and F).

### KLF2 controls an LCM gene signature induced by the serous cavity environment

**Figure 3.**
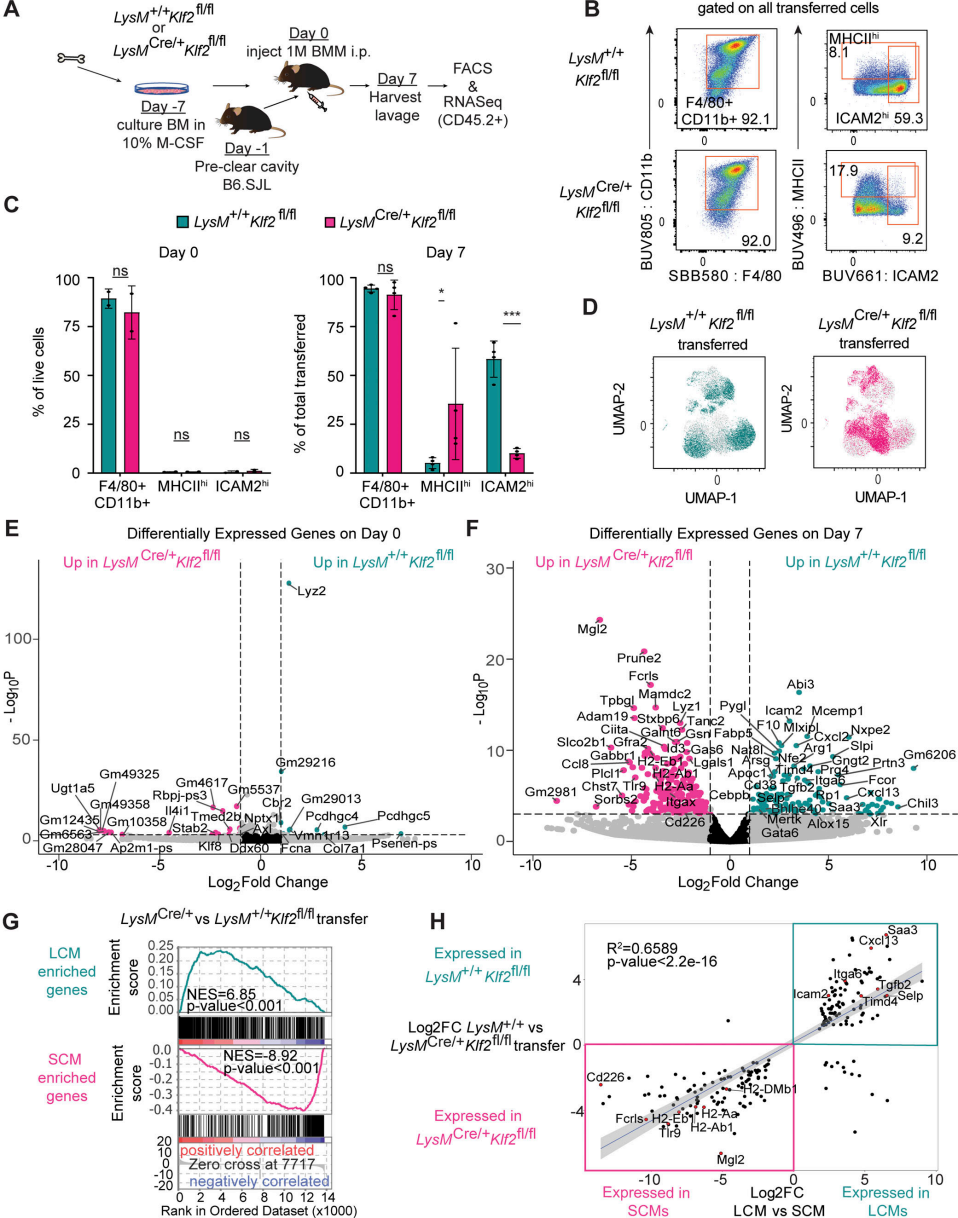
**KLF2 is required for macrophages to acquire expression of LCM identity genes. (A)** Schematic of experimental design for transfer of BMMs into the peritoneal cavity of recipient mice for subsequent harvest and analysis 7 days later. **(B and C)** Representative flow cytometry analysis (B) and compiled results (C) of *LysM*^*+/+*^*Klf2*^*fl/fl*^ or *LysM*^*Cre/+*^*Klf2*^*fl/fl*^ BMMs recovered on day 7 after transfer or prior to transfer (day 0). Data in B are gated on live, single, CD45.2^+^ cells. **(D)** Dimensionality reduction via UMAP of *LysM*^*+/+*^*Klf2*^*fl/fl*^- or *LysM*^*Cre/+*^*Klf2*^*fl/fl*^-transferred BMMs gated on live, single, CD45.2^+^ cells from four mice, downsampled to equal cells per genotype, barcoded, and concatenated. The projection was then overlayed with supervised gates based on genotype. **(E and F)** Volcano plots of DEG comparing *LysM*^*+/+*^*Klf2*^*fl/fl*^ to *LysM*^*Cre/+*^*Klf2*^*fl/fl*^ BMMs before (E) or after (F) transfer. Genes with a log_2_ fold change >|1| are colored grey. Genes with a P value of −log_10_ (P value) >4 are above the horizontal black line. Genes meeting both criteria are colored: teal, enriched in *LysM*^+/+^*Klf2*^*fl/fl*^; or pink, enriched in *LysM*^*Cre/+*^*Klf2*^*fl/fl*^. Genes meeting neither criterion are colored black. **(G)** GSEA of unfiltered expressed genes between day 7 transferred *LysM*^*+/+*^*Klf2*^*fl/fl*^ BMMs relative to *LysM*^*Cre/+*^*Klf2*^*fl/fl*^ BMMs. Gene sets for LCMs or SCMs were generated from DEG analysis of sorted WT LCMs or SCMs relative to each other. **(H)** Scatter plot of RNA-Seq expression data presenting the values of log_2_ fold change for shared DEGs in sorted small versus LCM (x axis) versus the DEGs between the day 7 sorted *LysM*^*+/+*^*Klf2*^*fl/fl*^ versus *LysM*^*Cre/+*^*Klf2*^*fl/fl*^ transferred BMMs (y axis). Data in B are from one experiment representative of three independent experiments; data in C and D are combined from three independent experiments, *LysM*^*+/+*^*Klf2*^*fl/fl*^ (*n* = 4), *LysM*^*Cre/+*^*Klf2*^*fl/fl*^ (*n* = 4) on day 0 and *LysM*^*+/+*^*Klf2*^*fl/fl*^ (*n* = 4), *LysM*^*Cre/+*^*Klf2*^*fl/fl*^ (*n* = 4) on day 7. Significance determined by ordinary two-way ANOVA with multiple comparisons and Šidák’s correction. Asterisks denote: ***P = 0.0006 and *P = 0.033. Sequencing (E–H) was performed once with *LysM*^*+/+*^*Klf2*^*fl/fl*^ (*n* = 3), *LysM*^*Cre/+*^*Klf2*^*fl/fl*^ (*n* = 3) on day 0, *LysM*^*+/+*^*Klf2*^*fl/fl*^ (*n* = 3), and *LysM*^*Cre/+*^*Klf2*^*fl/fl*^ (*n* = 3) on day 7. FC; fold change.

Tissue-resident macrophage populations from different tissues have distinct transcriptional profiles, and macrophages transferred into tissues can adopt the transcriptional state of that new environment (Lavin et al., 2014; Roberts et al., 2017). The inability of progenitors to differentiate into LCMs in the absence of KLF2 suggests that KLF2 may control expression of specific genes required for LCM differentiation or maintenance in the serous cavities. However, the complete loss of LCMs in *LysM*^*Cre/+*^*Klf2*^*fl/fl*^ mice prevented any direct analysis of gene expression at homeostasis, so we elected to transfer bone marrow macrophages (BMMs) into the peritoneal cavity, as a number of groups have previously shown that such cells receive tissue cues and adopt characteristics of LCMs (Lavin et al., 2014; Roberts et al., 2017; Sakai et al., 2019). *LysM*^*+/+*^*Klf2*^*fl/fl*^ or *LysM*^*Cre/+*^*Klf2*^*fl/fl*^ BMMs were transferred into congenitally marked hosts whose cavities were precleared by survival lavage to generate an open niche (Fig. 3 A). After 7 days, transferred *LysM*^*+/+*^*Klf2*^*fl/fl*^ BMMs (Fig. S2 E) had high expression of ICAM2 and low expression of MHCII, consistent with LCMs (Fig. 3, B and C). In contrast, *LysM*^*Cre/+*^*Klf2*^*fl/fl*^ BMMs showed higher expression of MHCII and lower expression of ICAM2. We also performed dimensionality reduction analysis of high-parameter flow cytometry data from the post-transfer BMMs and found that *LysM*^*+/+*^*Klf2*^*fl/fl*^ and *LysM*^*Cre/+*^*Klf2*^*fl/fl*^ cells separated into distinct clusters (Fig. 3 D). Thus, the expression of KLF2 contributes to the ability of transferred BMMs to upregulate known phenotypic markers of LCMs, and in the absence of KLF2 they phenotypically resemble transitional or SCMs.

To examine how lack of KLF2 impacted global gene expression in macrophages responding to tissue-derived signals, we sorted *LysM*^*+/+*^*Klf2*^*fl/fl*^ and *LysM*^*Cre/+*^*Klf2*^*fl/fl*^ BMMs 7 days after transfer and performed bulk RNA sequencing (RNA-Seq). On the day of transfer (day 0), *LysM*^*+/+*^*Klf2*^*fl/fl*^ BMMs and *LysM*^*Cre/+*^*Klf2*^*fl/fl*^ BMMs differed in expression by only 38 genes, none of which were SCM or LCM signature genes (Fig. 3 E). After 7 days in the cavity, we identified >594 significantly differentially expressed genes (DEGs) (fold change >2, false discovery rate <0.1) when comparing transferred *LysM*^*+/+*^*Klf2*^*fl/fl*^ or *LysM*^*Cre/+*^*Klf2*^*fl/fl*^ cells (Fig. 3 F). Transferred *LysM*^*+/+*^*Klf2*^*fl/fl*^ BMMs expressed higher levels of many LCM identity genes, including *Icam2*, *Timd4*, *Cebpb*, *Bhlhe40*, *Mertk*, *Cxcl13*, *Alox15*, and *Gata6*. *LysM*^*Cre/+*^*Klf2*^*fl/fl*^ BMMs expressed relatively higher levels of genes previously identified in SCMs, including MHCII genes (*H2-Aa*, *H2*-*Ab1*, *H2-Eb1*, and *Ciita*), *Itgax*, *Tlr9*, and *Cd226* (DNAM-1).

To quantify the enrichment of LCM or SCM genes in the DEG dataset generated by comparing the day 7 *LysM*^*+/+*^*Klf2*^*fl/fl*^ and *LysM*^*Cre/+*^*Klf2*^*fl/fl*^ BMMs, we performed preranked gene set enrichment analysis (GSEA). We generated two custom gene sets consisting of the significant DEGs between sorted WT LCMs and SCMs: one gene set with 643 LCM-enriched genes and one gene set with 381 SCM-enriched genes (Table S1). We then compared these gene sets with the 13,801 genes expressed between the two transferred BMM populations (Table S2), ranked by log_2_ fold change with 1,000 permutations in the classic enrichment score mode. The LCM gene set was strongly positively correlated with genes preferentially expressed by *LysM*^*+/+*^*Klf2*^*fl/fl*^ BMMs (P value <0.001, normalized enrichment score = 6.8), while the SCM gene set negatively correlated with genes expressed by the *LysM*^*+/+*^*Klf2*^*fl/fl*^ BMMs (P < 0.001, normalized enrichment score = −8.9) (Fig. 3 G). As the DEG comparison was between *LysM*^*+/+*^*Klf2*^*fl/fl*^ and *LysM*^Cre/+^*Klf2*^*fl/fl*^ BMMs, the SCMs gene set therefore strongly correlated with genes preferentially expressed by *LysM*^*Cre/+*^*Klf2*^*fl/fl*^ cells.

We also directly compared the expression level of shared DEGs between LCMs and SCMs and the *LysM*^*+/+*^*Klf2*^*fl/fl*^ and *LysM*^*Cre/+*^*Klf2*^*fl/fl*^-transferred BMMs (Fig. 3 H). This analysis revealed a striking correlation between DEGs in *LysM*^*+/+*^*Klf2*^*fl/fl*^ versus *LysM*^*Cre/+*^*Klf2*^*fl/fl*^ BMMs and DEGs in LCMs versus SCMs (adjusted R^2^ = 0.6589, F statistic = 391.2, P < 2.2e-16). Taken together, these data support a model in which KLF2 activity is induced by cavity-dependent signals and is required for the expression of genes associated with LCM identity.

### Identification of KLF2 target genes involved in LCM identity

**Figure S3.**
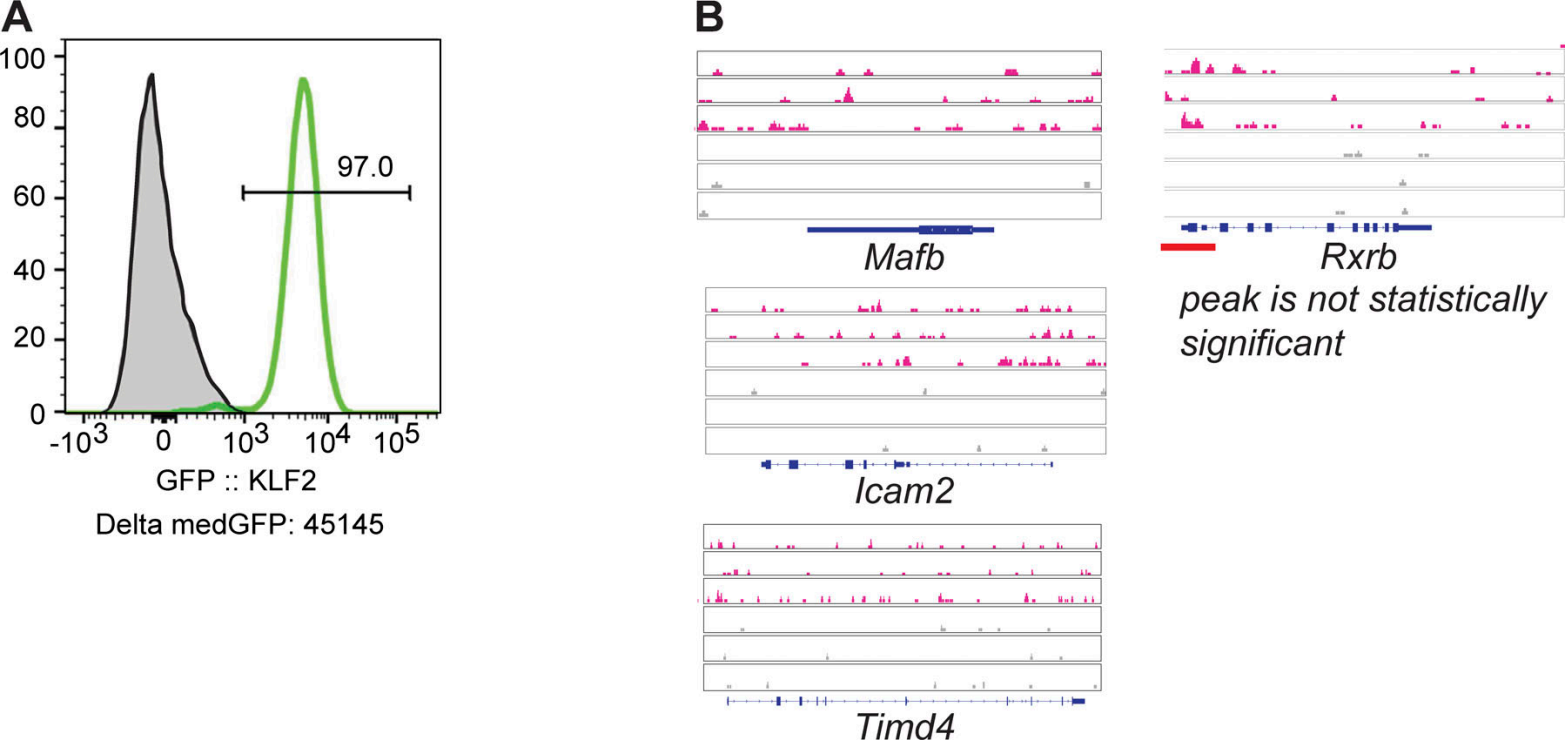
**KLF2 expression and gene regulation of surface markers in LCMs. (A)** Representative flow cytometry of GFP expression in LCMs (gated on live, single cells, CD3^−^CD19^−^B220^−^F4/80^+^CD11b^+^DNAM1^−^MHCII^−^ICAM2^+^) from GFP-KLF2 mice. **(B)** Genome browser tracks of anti-GPFKLF2 (pink) or IgG control (grey) CUT&RUN peaks from selected LCM genes.

To identify which of the KLF2-dependent genes are directly regulated by KLF2, we performed CUT&RUN on LCMs (Skene and Henikoff, 2017). To enhance the ability to pull down KLF2-specific fragments, we used an anti-GFP antibody on LCMs from GFP-KLF2 transgenic mice (Weinreich et al., 2009). These mice have an N-terminal eGFP knocked into the endogenous KLF2 locus, resulting in the expression of a fusion protein (Fig. S3 A). Additionally, the lower cell number requirement for CUT&RUN allowed us to generate biological replicates, further enhancing our ability to identify independently repeatable peaks with higher sensitivity as compared with ChIP-Seq. After filtering and trimming low-quality bases, reads were mapped and peak analysis was performed with SEACR (sparse enrichment analysis for CUT&RUN) (Meers et al., 2019). Mapped reads were normalized to spike-in *Escherichia coli* genome, and we are reporting here the top 1% of those peaks.

**Figure 4.**
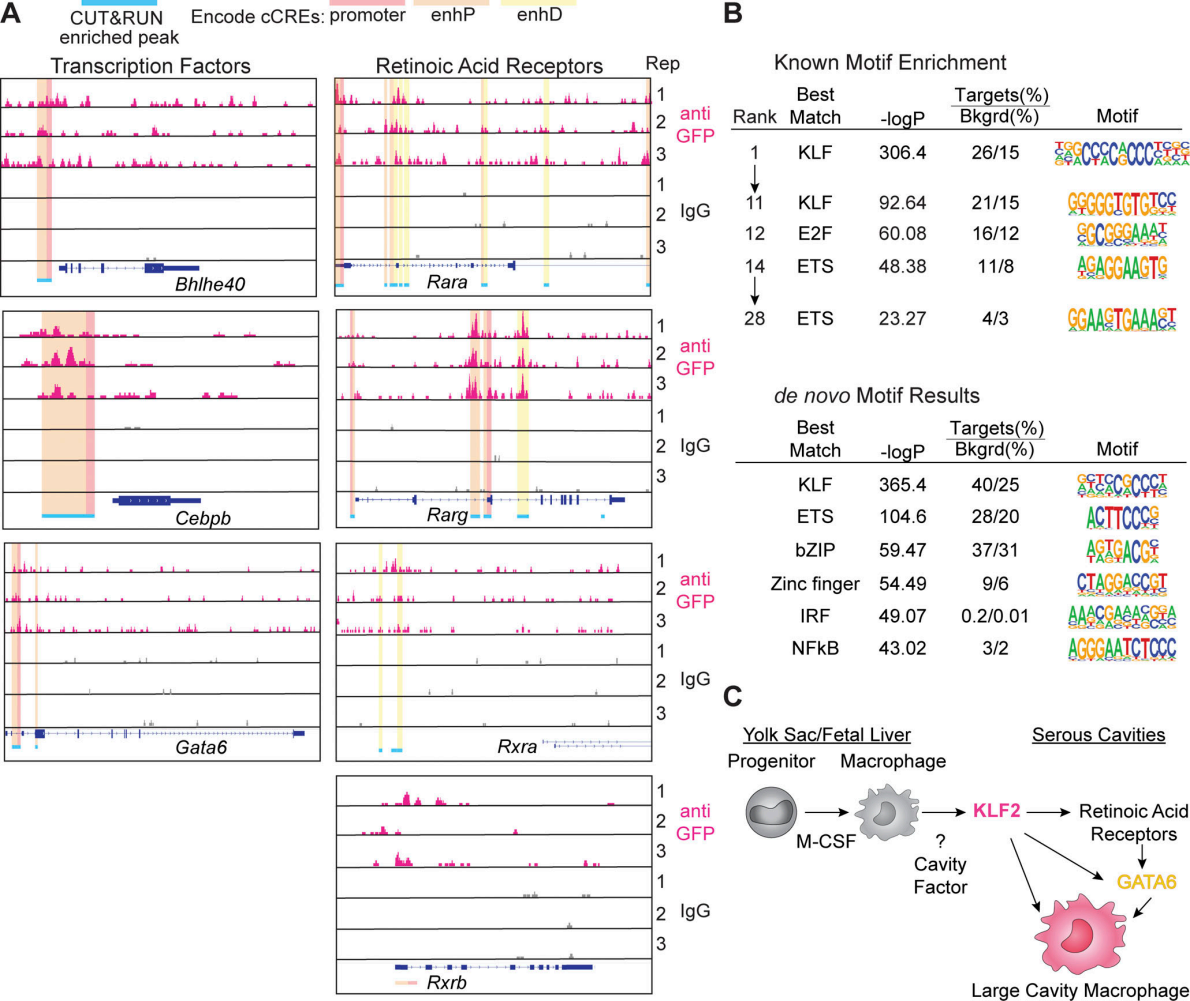
**Lineage determining factors are directly regulated by KLF2 in LCMs. (A)** Genome browser tracks of three independent samples showing anti-GFP KLF2 (pink) or IgG control (grey) CUT&RUN peaks from LCMs at the indicated genetic loci. Significant peaks (representing the top 1% of peak regions identified by SEACR) are indicated by blue bars. ENCODE cCREs (candidate cis-regulatory elements) are highlighted to indicate regulatory features that overlap with the peak region (red: promoter; orange: proximal enhancer; yellow: distal enhancer). All genes are shown 5′ → 3′, and peak signals of 0–10 are shown on the y axis. **(B)** Known motif enrichment and de novo motif analysis of LCM KLF2 CUT&RUN peaks compared with the IgG control. The top ranked KLF and ETS matches are shown along with the significance score (−logP) and enrichment of each motif, indicated as frequency of the motif within GFP peaks (target) relative to frequency of the motif within IgG peaks (Bkgrd). **(C)** Model of proposed function of KLF2 in LCMs. Data in A are from three samples of total LCMs (F4/80^+^CD11b^+^MHCII^−^DNAM-1^−^) split in half for anti-GFP or IgG control.

We found that KLF2 was bound to sites within or adjacent to several key LCM identity genes, and nearly all of these loci overlapped with ENCODE-identified candidate cis-regulatory elements (cCREs; predominately proximal enhancer and promoter regions) (Consortium et al., 2020). The genes bound by KLF2 included LCM transcription factors (*Bhlhe40*, *Cebpb*, and *Gata6*) (Fig. 4 A), but we did not detect statistically significant peaks in other LCM genes, including *Timd4*, *Icam2*, or *Mafb*, although there was KLF2 binding above background at these loci (Fig. S3 B). Additionally, KLF2 peaks were detected in genes encoding the retinoic acid receptors (*Rara*, *Rarg*, and *Rxra*) (Fig. 4 A), confirming the importance of KLF2 in initiating the broader transcriptional identity of LCMs.

Performing transcription factor motif analysis on the 1% of identified peaks revealed that the top 11 enriched known motifs all matched to KLF-binding sites, confirming the validity of the data with additional representation of E2F-binding sites and ETS transcription factor motifs (Fig. 4 B). When we performed de novo motif discovery on total enriched peaks, KLF-binding sites were the most enriched followed by ETS, with lesser contribution from sites for bZIP, zinc finger, IRF, and NFκB transcription factors (Fig. 4 B). Critically, genes directly bound by KLF2 were not co-enriched for GATA6 or RAR/RXR-binding motifs, consistent with our model that KLF2 is required for the responsiveness of developing LCMs to retinoic acid (Fig. 4 C). These analyses demonstrate the importance of KLF2 for direct regulation of the expression of known LCM transcription factors (i.e., GATA6 and RARs) while also potentially collaborating with general macrophage transcription factors like PU.1 (ETS) and AP-1/ATF/CEBP (bZIP). The paramount role of KLF2 in the transcriptional network controlling LCM identity (Fig. 4 C) likely explains the more severe effect on LCM development observed with KLF2 deficiency relative to Bhlhe40 deficiency (Jarjour et al., 2019; Rauschmeier et al., 2019), C/EBPβ deficiency (Cain et al., 2013; Dorr et al., 2022), or GATA6 deficiency.

### Ectopic expression of KLF2 induces LCM identity in vitro

**Figure 5.**
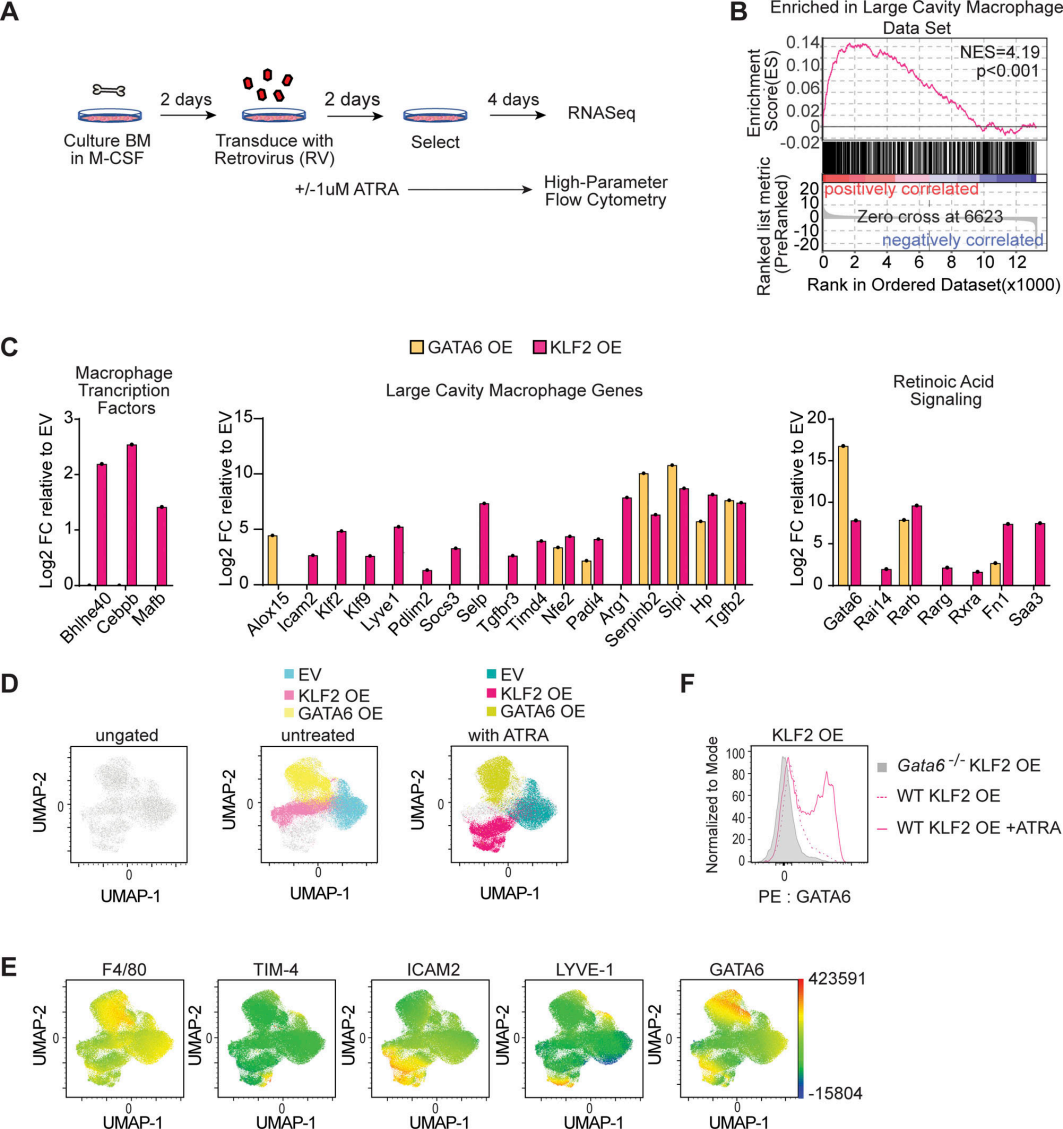
**Ectopic expression of KLF2 confers LCM phenotype and transcriptional identity in vitro. (A)** Graphical representation of experimental design for the RV OE of transcription factors in in vitro–derived BMMs. **(B)** GSEA of unfiltered expressed genes between KLF2 OE BMMs and EV BMMs. Gene sets for LCMs and SCMs are derived from DEG analysis of sorted WT LCMs or SCMs relative to each other. **(C)** Bar graphs depicting the log_2_ fold change of RNA-Seq data from transcription factor–transduced BMMs relative to EV of the indicated LCM relevant genes. **(D)** Dimensionality reduction via UMAP of live, single, virus+ cells from EV, KLF2 OE, or GATA6 OE cells and downsampled to normalize cells per genotype, which were barcoded and concatenated. The left panel depicts all concatenated events, the middle panel shows the overlay of untreated cells transduced as indicated, and the right panel shows the overlay of ATRA treated cells transduced as indicated. **(E)** UMAP plots generated as described in D showing expression intensities of the indicated markers. **(F)** GATA6 expression measured by intracellular staining and flow cytometry of the indicated BMM genotypes transduced with KLF2 OE RV with or without ATRA treatment. RNA-Seq was performed on samples generated in three independently performed transduction experiments (B and C). Data are from one experiment representative of three independent experiments (D–F). FC; fold change.

**Figure S4.**
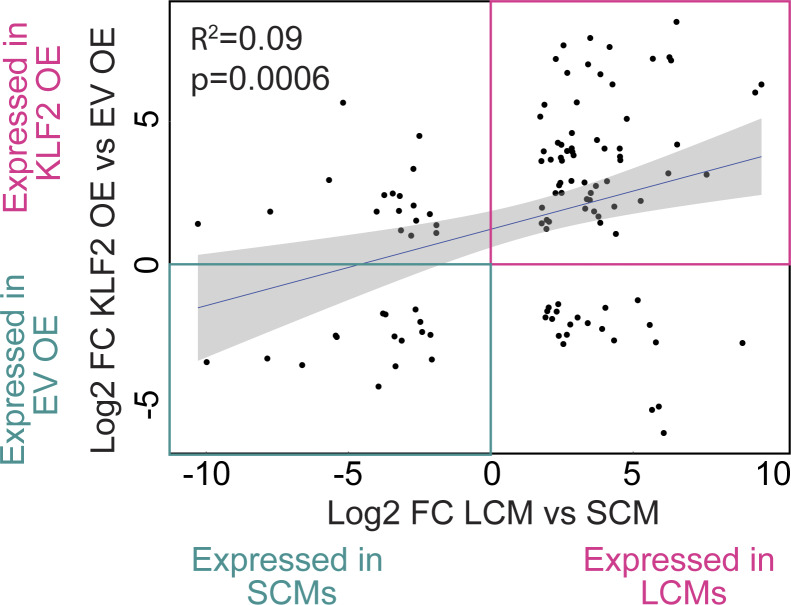
**Similarity of shared DEGs in KLF2 OE BMMs and LCMs.** Scatter plot of RNA-Seq expression data presenting the values of log_2_ fold change for shared DEGs in sorted LCMs versus SCMs (x axis) versus the DEGs between the EV versus KLF2-transduced BMMs (y axis).

Given that KLF2 regulates so many of the genes involved in sensing cavity-dependent signals (Figs. 3 and 4), we employed a simplified model of overexpression (OE) by retroviral (RV) transduction in in vitro–derived BMMs (Fig. 5 A) to examine whether KLF2 can drive expression of LCM genes in the absence of the cavity environment. We first analyzed what transcripts were induced by the expression of KLF2 or GATA6 relative to the empty vector (EV) control by bulk RNA-Seq. Similar to our analysis of transferred BMMs, we compared the DEGs from the comparison of KLF2 OE to the EV control with DEGs between LCMs and SCMs. Strikingly, OE of KLF2 alone, without any additional tissue-specific soluble factors or any cavity-resident cells, was able to recapitulate similar induction of many of the shared DEGs in LCMs but not SCMs (adjusted R-squared: 0.09108 F-statistic: 12.62 on 1 and 115 degrees of freedom, P value: 0.0005532) (Fig. S4). To quantify this similarity, we performed GSEA using the LCMs gene set from Fig. 3 and observed a strong positive correlation between the LCM gene set and genes preferentially expressed in the KLF2 OE BMMs relative to EV control BMMs (P value <0.001, normalized enrichment score = 4.2) (Fig. 5 B).

Notably, OE of KLF2, but not GATA6, induced the expression of LCM transcription factors, including *Bhlhe40*, *Mafb*, and *Cebpb* (Fig. 5 C). KLF2 also induced many canonical LCM identity genes. A subset of these genes was induced only by KLF2 (e.g., *Icam2*, *Lyve1*, *Pdlim2*, and *Socs3*) (Fig. 5 C), while others were induced by both KLF2 and GATA6 (e.g., *Serpinb2*, *Hp*, and *Tgfb2*). The differences in expression between these two groups of genes is most likely due to the fact that KLF2 also induced the expression of retinoic acid receptors (*Rarb*, *Rarg*, and *Rxra*) and *Gata6* (Fig. 5 C), which can in turn induce expression of retinoic acid–dependent LCM identity genes (e.g., *Serpinb2*, *Hp*, and *Tgfb2*). Thus, KLF2 expression is sufficient to induce the expression of many LCM identity genes, including the retinoic acid/GATA6 axis previously identified as critical for the specification and function of these cells.

To further test the hypothesis that KLF2 OE induces a macrophage state that more closely recapitulates LCMs than GATA6 OE, we used high-dimensional flow cytometry. Transduced cells were cultured in the presence or absence of exogenously added all-trans retinoic acid (ATRA) to confirm the functional importance of induced retinoic acid receptor expression and to determine if exogenous ATRA could enhance adoption of an LCM-like phenotype. When we concatenated equal events from all conditions and performed dimensionality reduction via UMAP, the three transduced BMM populations clustered separately from each other, indicating that OE of KLF2 or GATA6 distinctly altered the phenotype of BMMs (Fig. 5 D). The addition of ATRA changed the KLF2 OE transductants but had little effect on the EV or GATA6 OE cells (Fig. 5 D), consistent with GATA6 being downstream of retinoic acid signaling (Buechler et al., 2019; Okabe and Medzhitov, 2014). When we examined known LCM marker expression, KLF2 OE cells were the predominate population that gained expression of markers (ICAM2, LYVE-1, and TIM-4) that phenotypically define LCMs or transitional LCMs (Finlay et al., 2023; Han et al., 2024). The expression of these markers was further enhanced by ATRA only in KLF2 OE BMMs (Fig. 5 E), suggesting that KLF2 expression precedes LCM identity generation, including the ability to receive retinoic acid signals (Fig. 4 C). Detection of GATA6 protein by intracellular staining confirmed induction in KLF2 OE cells, which was further enhanced by addition of ATRA (Fig. 5 F). Together these RNA-Seq and high-parameter flow cytometry analyses show that the expression of KLF2 and the addition of ATRA synergize to induce and enhance expression of LCM markers in vitro.

### AM identity requires KLF4

**Figure 6.**
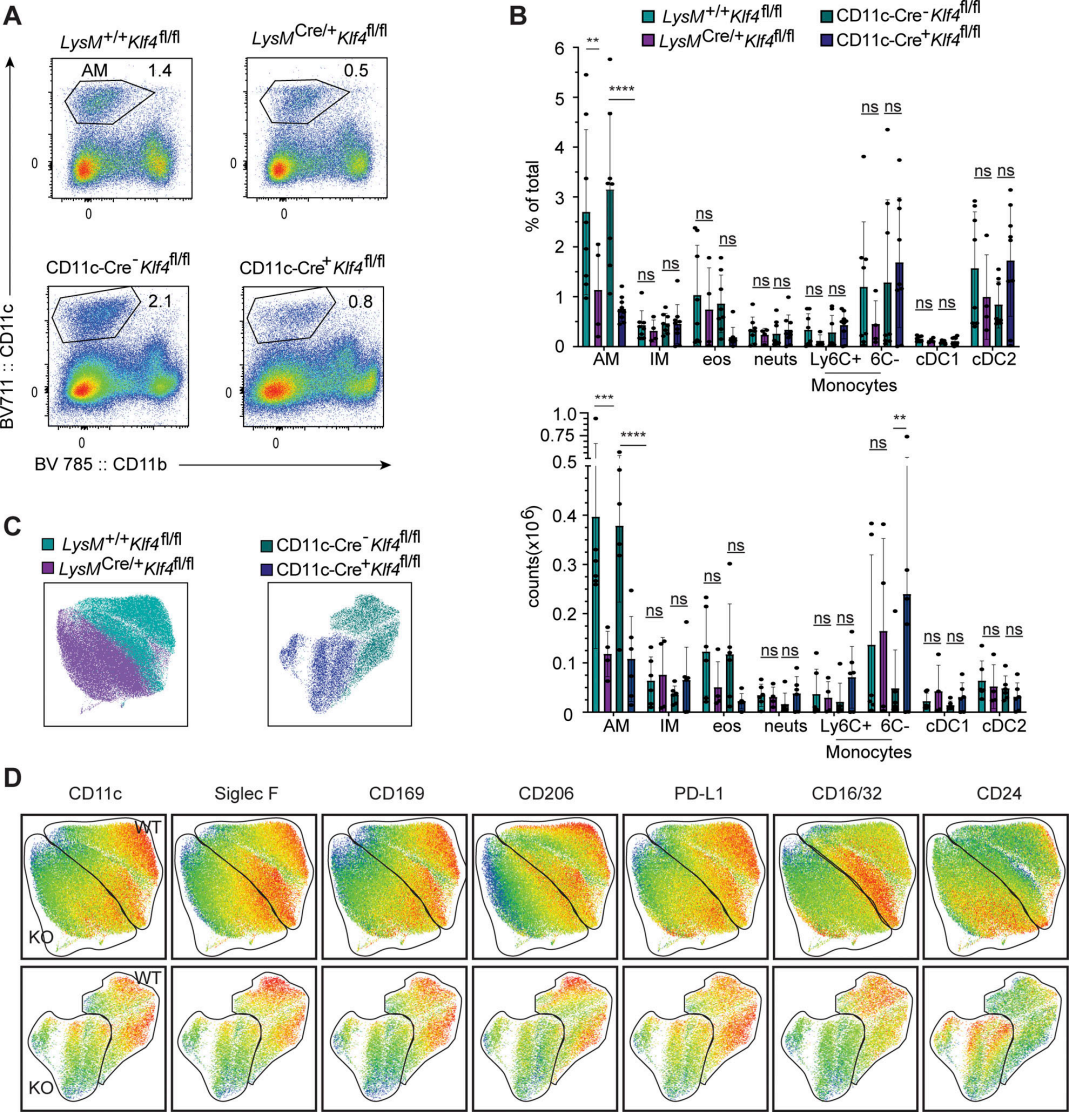
**KLF4 is required for AM development and identity. (A)** Flow cytometry of cells in lungs from the indicated mice, gated on live, single, CD45^+^CD3^−^B220^−^CD19^−^ cells. Numbers adjacent to gates indicate percentage of total live events for each gate. **(B)** Bar graphs depicting the percentage number of total live cells of the following lung populations (representative gates show in in Fig. S2 B) as measured by flow cytometry: CD11c^+^CD11b^−^Siglec-F^+^CD24^−^ (AMs), CD11b^+^MHCII^+^CD24^−^CD64^+^ (interstitial macrophages, IM), MHCII^−^CD11b^+^CD24^+^Siglec-F^+^ (eosinophils, eos), MHCII^−^CD11b^+^CD24^+^Ly6G^+^ (neutrophils, neuts), MHCII^−^CD11b^+^CD64^+^Ly6C^+^ or Ly6C^mid/−^ (monocytes), CD11c^+^CD11b^−^Siglec-F^−^CD24^+^ (cDC1), and CD11b^+^MHCII^+^CD24^+^CD64^−^ (cDC2). **(C)** Dimensionality reduction via UMAP of AMs was performed after downsampling to equivalent cells per genotype, barcoding, and concatenation. The resulting projection is color coded to distinguish WT from Klf4-deficient cells. **(D)** Heatmaps of defining marker expression for AMs from LysM-Cre (top) or CD11c-Cre (bottom) mice overlaid onto the UMAP projections from C. The WT or KO cell clusters based on C are indicated. Data in A are representative of four independent experiments. Data in B are combined from four independent experiments. Data in C and D are combined from three experiments. Significance determined by ordinary two-way ANOVA with multiple comparisons and Šidák’s correction; Asterisks denote: ****P = < 0.0001, ***P = 0.0006, and **P = 0.0021.

**Figure S5.**
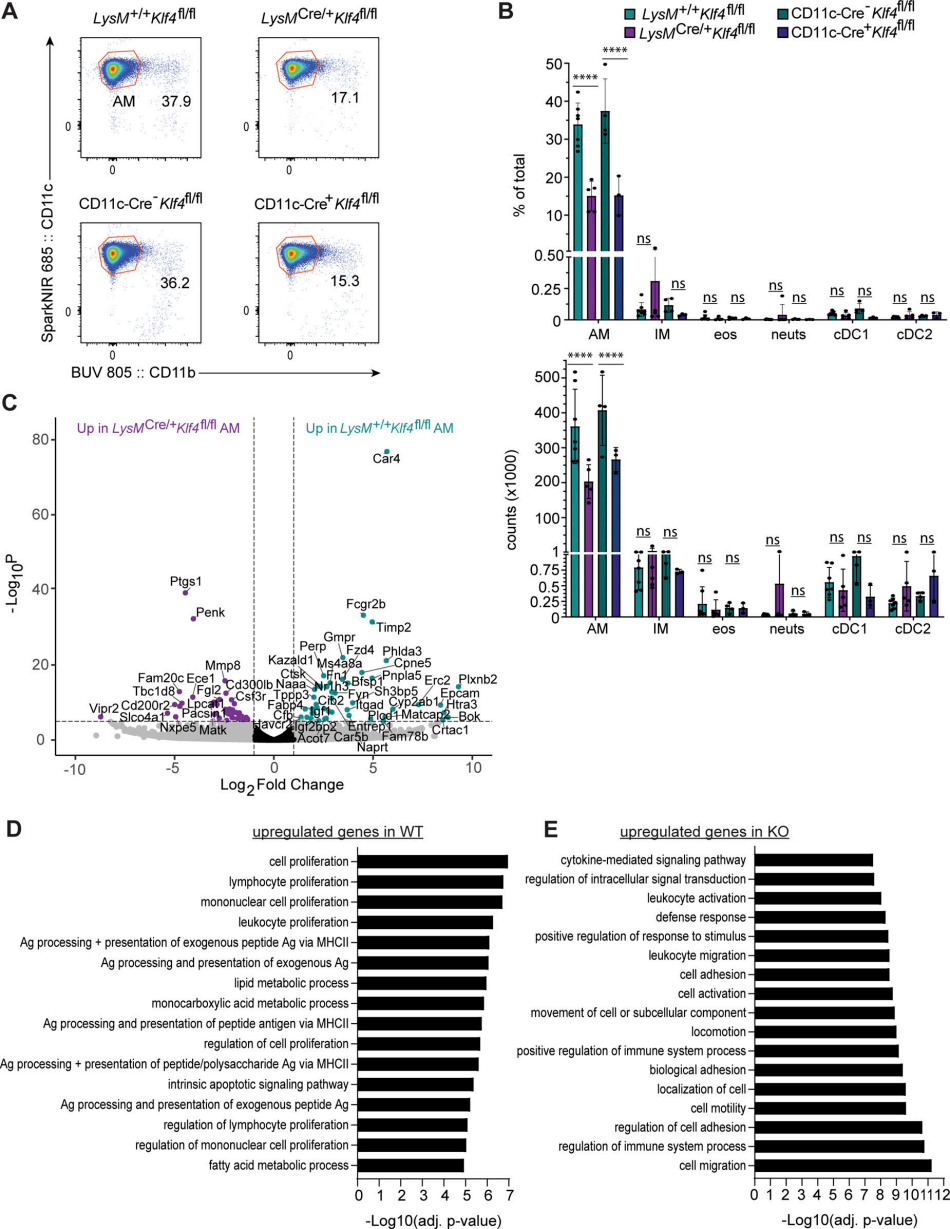
**KLF4 dependency in BALF AM development and gene expression in AMs. (A)** Representative flow cytometry of BALF AMs (live, single, CD19^−^CD11c^+^CD11b^−^SiglecF^+^CD24^−^) from the indicated mice. Numbers adjacent to gates indicate the percentage of total live events for each gate. **(B)** Bar graphs depict the percent of total and total number of the following BALF populations (representative gates shown in Fig. S2 B): CD11c^+^CD11b^−^SiglecF^+^CD24^−^ (AMs); CD11b^+^MHCII^+^CD24^−^CD64^+^ (interstitial macrophages, IM); MHCII^−^CD11b^+^CD24^+^SiglecF^+^ (eosinophils, eos); MHCII^−^CD11b^+^CD24^+^Ly6G^+^ (neutrophils, neuts); CD11c^+^CD11b^−^SiglecF^−^CD24^+^ (cDC1); and CD11b^+^MHCII^+^CD24^+^CD64^−^ (cDC2). **(C)** Volcano plot of expressed genes comparing sorted *LysM*^*+/+*^*Klf4*^*fl/fl*^ to *LysM*^*Cre/+*^*Klf4*^*fl/fl*^ AM. Genes with a log_2_ fold change >|1| are colored grey. Genes with a P value of −log_10_ (P value) >6 are above the horizontal black line. Genes meeting both criteria are colored: teal, enriched in *LysM*^*+/+*^*Klf4*^fl/fl^; purple, enriched in *LysM*^*Cre/+*^*Klf4*^*fl/fl*^. Genes meeting neither criterion are colored black. **(D)** Top 25 GO term analysis results of genes upregulated in *LysM*^*+/+*^*Klf4*^*fl/fl*^ AMs. **(E)** Top 25 GO term analysis results of genes upregulated in *LysM*^*Cre/+*^*Klf4*^*fl/fl*^ AMs. Bulk RNA-Seq (E) was performed from one set of samples containing two biological replicates of each genotype. Asterisks denote: ****P < 0.0001.

Next, we considered whether the requirement for KLF2 in LCMs reflects a more general function of KLFs in regulating the development and identity of resident macrophage populations in other tissues. In a previous study comparing gene expression in embryonic and neonatal macrophages across tissues, KLF4 was shown to be preferentially induced in embryonic and neonatal AMs when compared with brain, liver, kidney, and epidermis macrophages, similar to the expression of AM identity genes *Pparg* and *Cebpb* (Mass et al., 2016). Taking this into account and because we did not observe any defect in AMs in *LysM*^*Cre/+*^*Klf2*^*fl/fl*^ mice or any contribution from KLF2-deficient donor cells to AMs in radiation chimeras, we considered whether KLF4 might fulfill a role in AMs similar to that of KLF2 in LCMs. Due to the previously described difference in the ability of *LysMCre* and *CD11cCre* to target genes in AMs (Schneider et al., 2014), we crossed both Cre drivers to *Klf4*^*fl/fl*^ mice to ensure rigor in our analyses. Analyses of AMs and other lung myeloid cells in *LysM*^*Cre/+*^*Klf4*^*fl/fl*^ and *CD11c-Cre*^*+*^*Klf4*^*fl/fl*^ mice revealed a specific reduction in both frequency and number of AMs, while leaving other myeloid populations relatively unaffected (Fig. 6, A and B). We observed the same phenomena in cells recovered from bronchoalveolar lavage (Fig. S5, A and B). Dimensionality reduction analysis and unsupervised cluster identification of high-parameter flow cytometry data of AMs also revealed distinct clustering of the WT versus KLF4-deficient AMs for both Cre drivers, indicating that the remaining AMs in the KLF4-deficient lines were altered relative to WT cells (Fig. 6 C). Indeed, KLF4-deficient AMs from *LysM*^*Cre/+*^*Klf4*^*fl/fl*^ and *CD11c-Cre*^*+*^*Klf4*^*fl/fl*^ mice expressed lower levels of CD11c, SiglecF, CD169, CD206, PD-L1, and CD16/32 and higher levels of CD24 (Fig. 6 D).

**Figure 7.**
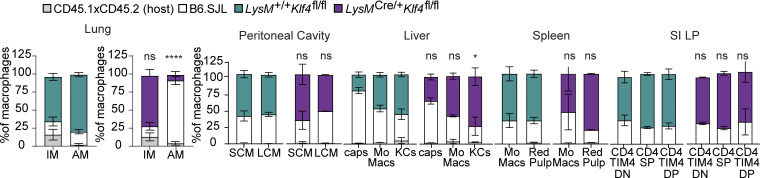
**The requirement for KLF4 in AM development is cell intrinsic.** Frequency of cells of the indicated genotypes within lung AMs, interstitial macrophages, and monocyte and resident macrophage subsets in other tissues from radiation chimeras generated with *LysM*^+/+^*Klf4*^fl/fl^:B6.SJL or *LysM*^Cre/+^*Klf4*^fl/fl^:B6.SJL mixtures of donor bone marrow into CD45.1 × CD45.2 hosts. Data in A and B are from one experiment representative of two independent experiments and presented as mean with SD of three to four chimeric animals per group. Significance determined by Fisher’s exact test of relative contributions of each genotype in B6.SJL:*LysM*^Cre/+^*Klf4*^fl/fl^ chimeras compared with B6.SJL:*LysM*^+/+^*Klf4*^fl/fl^ chimeras. Asterisks denote: ****P < 0.0001 and *P = 0.033.

To address whether the reduction in AMs in KLF4-deficient animals was the result of a cell-intrinsic defect in the AMs themselves, we made radiation bone marrow chimeras using mixed *LysM*^*+/+*^*Klf4*^*fl/fl*^ and *LysM*^*Cre/+*^*Klf4*^*fl/fl*^ donor bone marrow. As controls, we included radiation chimeras with mixtures of congenitally distinct WT bone marrow. Analysis of AMs from these chimeras revealed a striking skewing toward *LysM*^*+/+*^*Klf4*^*fl/fl*^ cells over *LysM*^*Cre/+*^*Klf4*^*fl/fl*^ cells, while both genotypes were equally represented among the monocyte-derived interstitial macrophage population of the lung (Fig. 7). This defect is similar to what has been reported for PPARγ-deficient or C/EBPβ-deficient cells in similar mixed bone marrow chimera analyses (Dorr et al., 2022; Schneider et al., 2014). Importantly, KLF4 deficiency did not have any major impact on macrophage subsets in spleen, liver, or small intestine (Fig. 7). Overall, these results support a cell-intrinsic role for KLF4 in AMs, consistent with our analysis of *LysM*^*Cre/+*^*Klf4*^*fl/fl*^ and *CD11c-Cre*^*+*^*Klf4*^*fl/fl*^ mice.

Finally, to evaluate more comprehensively the impact of KLF4 deficiency on AMs, we sorted AMs from *LysM*^*+/+*^*Klf4*^*fl/fl*^ and *LysM*^*Cre/+*^*Klf4*^*fl/fl*^ mice and performed RNA-Seq. Comparison of DEGs revealed that a number of known AM identity genes were enriched *LysM*^*+/+*^*Klf4*^*fl/fl*^ AMs relative to *LysM*^*Cre/+*^*Klf4*^*fl/fl*^ AM, including *Car4*, *Perp*, *Epcam*, *Cpne5*, *Fcgr2b* (CD32), *Cd206*, *Cd74*, and *Ctsk* (Fig. S5 C). Gene Ontology (GO) enrichment analysis of the genes preferentially expressed in the *LysM*^+/+^*Klf4*^*fl/fl*^ AMs yielded terms related to cell proliferation, lipid, fatty acid and carboxylic acid metabolism, and antigen presentation via MHCII (Fig. S5 D). These pathways and processes have previously been recognized as important to AMs function and were also enriched in the differential gene signatures comparing WT AMs to AMs lacking PPARγ or C/EBPβ (Dorr et al., 2022). The *LysM*^*Cre/+*^*Klf4*^*fl/fl*^ AM had higher relative expression of genes related to cell adhesion and mobility as well as genes involved in positive regulation of the immune system and cytokine signaling (Fig. S5 E). The expression of these genes in *LysM*^*Cre/+*^*Klf4*^*fl/fl*^ AMs may indicate that these altered AMs represent newly arriving cells that are trafficking to the alveolar space due to the partially unfilled niche, similar to the increase in SCMs and transitional macrophages that we observe in the serous cavities in *LysM*^*Cre/+*^*Klf2*^*fl/fl*^ mice.

## Discussion

Tissue-resident macrophages develop during embryogenesis and are instructed through local signals to gain tissue-specific identities. Over the past decade, extensive work from many labs has begun to identify the key transcription factors and molecular pathways that are important for each resident macrophage population, but how such factors and pathways cooperate to determine a given macrophage identity remains unclear and is an area of intense investigation. In this study, we establish that members of the group 2 KLF family are critical for development of two specific tissue-resident macrophage populations.

Previous experiments have identified retinoic acid receptor signaling and GATA6 as essential transcriptional regulators of embryonically derived LCMs (Buechler et al., 2019; Casanova-Acebes et al., 2020; Finlay et al., 2023; Gautier et al., 2012, 2014; Okabe and Medzhitov, 2014; Rosas et al., 2014). Here we show, in a side-by-side comparison, that KLF2 deficiency has a more dramatic effect on the development of LCMs than GATA6 deficiency in a cell-intrinsic, tissue-specific manner. Through multiple lines of evidence, we determine that the induction of KLF2 is required when tissue residency is established and not during earlier stages of macrophage development. We show that KLF2 promotes the induction of other transcription factors important in LCMs, including GATA6, and enables increased capacity for retinoic acid sensing through direct regulation of retinoic acid receptors, which also induce GATA6 expression. Together, these data place KLF2 upstream of previously identified regulators of LCM development and demonstrate that KLF2 is sufficient to induce many of the critical genes required for LCM development.

KLF2 is necessary for retinoic acid-dependent elements of LCM identity but is also essential for many of the LCM genes that are retinoic acid independent, by both direct and indirect mechanisms (not all genes we observed as being transcriptionally regulated by KLF2 were also bound directly by KLF2 in our CUT&RUN). For example, reduced TLR signaling is a distinct module of LCM identity that is KLF2 dependent and retinoic acid independent (Roberts et al., 2017). Though retinoic acid signaling and GATA6 are important for several identified LCM functions (Casanova-Acebes et al., 2020; Deniset et al., 2019; Finlay et al., 2023; Okabe and Medzhitov, 2014), *Gata6*- or retinoic acid receptor–deficient LCMs still express KLF2, are present in greater numbers than in *Klf2*-deficient mice, and have a fairly normal LCM phenotype.

While the cells responsible for retinoic acid production in the cavity have been identified (Buechler et al., 2019), it remains unknown how KLF2 expression is regulated in LCMs. *Klf2* expression is induced by laminar flow in endothelial cells or by blocking HMG-CoA reductase in endothelial and T cells via the induction of the transcription factor MEF2 (Ahmad and Lingrel, 2005; Maejima et al., 2014; Parmar et al., 2006; Villarreal et al., 2010). It is possible that macrophage progenitor cells in the cavity experience a specific flow rate or that the cavity induces a change in metabolism that activates MEF2, either through HMG-CoA accumulation or inhibition of cholesterol synthesis. The relevance for these potential mechanisms in LCMs will be important to address in future work. Of course, distinct undiscovered mechanisms may also be involved in KLF2 regulation in these cells.

Our work also suggests that KLF4, a different group 2 KLF, may play a similar role in AM development, although we do not provide a mechanistic link to downstream regulators of AM development as we do for KLF2 and LCMs. While the precise mechanisms of KLF4 function in these cells require further investigation, our results demonstrate that expression of many AM genes is altered in the absence of KLF4. Previous studies have demonstrated the importance of GM-CSF and TGF-β signaling to induce C/EBPβ and PPARγ expression in AMs (Dorr et al., 2022; Schneider et al., 2014; Yu et al., 2017). What regulates KLF4 in these cells and whether KLF4 regulates these previously identified pathways or other aspects of AM development remains unclear. It is intriguing that both subsets of macrophages we have identified as having group 2 KLF dependency also both depend on C/EBPβ. Because of this concordance, we speculate that KLF4 may also act upstream of C/EBPβ in AMs as KLF2 does in LCMs.

The requirement of individual KLF family members for development of specific tissue-resident macrophage populations is somewhat unexpected, especially considering their rather broad expression across many cell types. However, we contend that this characteristic may explain why their role in macrophage function was previously missed. When looking for transcriptional regulators that are critical for the identity of a particular macrophage population, investigators have focused on genes with narrow, population-specific expression. This approach may miss genes with broader expression that nevertheless play critical regulatory functions in particular cells. Another approach to identify transcription factors important for macrophage development is motif analysis of histone marker ChIP- or ATAC-Seq datasets. Through these types of experiments, KLF-binding sites have been noted to be enriched at genomic loci of active genes of various tissue-resident macrophages (Gosselin et al., 2014; Honda et al., 2022; Lavin et al., 2014; Sajti et al., 2020). However, KLF-binding sites were also enriched in the total enhancer regions of neutrophils and monocytes (Lavin et al., 2014), further supporting the idea that KLF regulation is a common feature of myeloid cells. Finally, this kind of computational analysis of open chromatin or enhancer regions can only identify KLF-binding motifs in the broadest sense (including in our own data, Fig. 4), as all KLFs share the same core-binding motif.

Finally, our data ascribe a new role for KLFs in macrophages that is different than what has been previously reported. Previous work has suggested that KLF2 and/or KLF4 limit the activation and proinflammatory functions of monocytes and macrophages within various tissue and in various disease models (Chakraborty et al., 2023; Das et al., 2006, 2012; Herta et al., 2021; Liao et al., 2011; Mahabeleshwar et al., 2011; Manoharan et al., 2019; Roberts et al., 2017; Shi et al., 2014; Sweet et al., 2020). While our analyses do not directly refute an anti-inflammatory role for KLF2 and KLF4, we do believe that such effects on activation may be secondary to the role that these transcription factors play in macrophage differentiation within tissues. For example, the inability of infiltrating KLF2-deficient monocytes to respond to tissue-derived cues in the inflamed serous cavities may result in activated monocyte-derived macrophages, but this gene signature may reflect the absence of KLF2-driven differentiation more than a direct regulation of macrophage activation.

In summary, we show that commonly expressed transcription factors belonging to the group 2 KLF family are required for the development of distinct tissue-resident macrophage populations. In LCMs, this requirement is based on KLF2-dependent expression of developmental regulators that enable cells to sense tissue-derived signals. Our results suggest that future work should consider potential roles for more broadly expressed transcription factors in development and identity of resident macrophages.

### Limitations of study

While our work provides genetic evidence for the importance of KLF4 in AM development, the defect is not as complete as the block in LCM development in the absence of KLF2. A lack of reagents prevented us from performing CUT&RUN analyses of KLF4 binding. Therefore, our evidence for KLF4 function in AMs lacks the mechanistic insight that we were able to dissect for KLF2 in LCMs. In addition, our studies are limited to macrophage populations in adult mice. We did not examine how deficiency in KLF2 and KLF4 impacts the ontogeny of embryonic and neonatal macrophages in cavities or lungs. Finally, we demonstrate a specific role for two KLF family members in two different tissue-resident macrophage populations, but enrichment of KLF motifs has been observed in other macrophage populations (e.g., Kupffer cells). Additional work will be required to address whether other KLF family members, especially those in group 2 (i.e., KLF1, KLF5, KLF6, and KLF7), play any role in other tissue macrophage populations.

## Materials and methods

### Mice

All animal procedures were approved by the University of California, Berkeley Institutional Animal Care and Use Committee in accordance with the University of California, Berkeley research guidelines for the care and use of laboratory animals. The following mice were used in this study: C57BL/6J (000664), B6.SJL (B6.Ptprc^a^Pep^c^/BoyJ, 002014), JaxBoy (C57BL/6J-Ptprcem6Lutzy/J, 033076), *LysM*^Cre^ (Lyz2Cretm1[Cre]Ifo, 004781), *Klf2*^*fl/fl*^ (gift from Jerry B. Lingrel, University of Cincinnati, Cincinnati, OH, USA; deceased), *LysM*^*Cre*^*Gata6*^*fl/fl*^ (gift from Paul Kubes, University of Calgary, Calgary, Canada), *LysM*^*Cre*^*Klf4*^*fl/fl*^ (floxed *Klf4* allele: MMRRC 29877), CX3CR1-Cre (Tg[Cx3cr1-cre]MW126Gsat/Mmucd, MMRRC: 036395-UCD), CD11c-Cre *Klf4*^flfl^ (Tg[Itgax-cre]1-1Reiz/J, 008068, cross generated in-house), GFP-KLF2 (B6[C]-Klf2tm1.1Khog/JmsnJ, gift from Stephen Jameson and Kristin Hogquist labs, University of Minnesota, Minneapolis, MN, USA), and CD45.1 × CD45.2 F1 (generated in our colony by crossing C57BL/6J to B6.SJL [B6.Ptprc]). Both male and female mice between 7 and 9 wk of age were used for all experiments, except for radiation bone marrow chimeric studies where mice were analyzed 8–12 wk after bone marrow transfer into 5–6-wk-old mice.

### Cell preparation for flow cytometry

Peritoneal lavage was obtained by injecting 5 ml PBS (Gibco) containing 2 mM EDTA (Thermo Fisher Scientific) and 2% FCS (Gibco or Omega) with a 23-gauge needle into the peritoneal cavity and then retrieving the solution back out with the same syringe and a 21-gauge needle. Pleural lavage is performed the same, but with 3 ml PBS containing 2 mM EDTA and 2% FCS.

The spleen, lung, and liver were harvested and homogenized into a single-cell suspension with C tubes on a GentleMacs (Miltenyi) with appropriate tissue programs. For spleen, 0.07 mg/ml LiberaseTM (Roche) and 5 µg/ml DNaseI (Sigma-Aldrich) were used in 2 ml HBSS with Ca/Mg (14025092; Gibco), and the same concentration was used for liver in 5 ml HBSS with Ca/Mg. For lung, 0.07 mg/ml LiberaseTM and 45 µg/ml DNaseI in 2 ml HBSS with Ca/Mg. Tissues were incubated for 20 min at 37°C and then finished on the GentleMacs. Enzymes were quenched with PBS with 2 mM EDTA and 2% FCS on ice, and then red blood cells were lysed with room temperature ACK Lysis Buffer (Gibco) and passed through a 100-μm cell strainer.

For intestinal lamina propria cell preparation, Peyer’s patches were removed with scissors, and the intestine was cut open longitudinally. The intestinal contents and mucus were removed by gentle scraping with forceps and rinsing with PBS. The intestine was cut into 1-cm pieces and incubated in HBSS buffer containing 1 mM dithiothreitol (Fisher Scientific) and 10% FCS at 37°C while stirring for 20 min to remove intestinal epithelial lymphocytes, which were discarded. Intestinal tissue was transferred to HBSS (Gibco) containing 1.3 mM EDTA (Thermo Fisher Scientific) and stirred at 37°C for 20 min to remove the epithelium, which was discarded. Remaining intestinal pieces were then incubated in HBSS containing 5% FBS and 150 U/ml collagenase type 2 (Worthington) at 37°C with stirring for 45 min to isolate LP cells. LP cells were further purified by gradient centrifugation at 2,800 rpm for 20 min with 44% and 67% Percoll (GE Healthcare/Cytiva) at room temperature. Afterward, the mucoid top layer of dead cells and epithelial cells was removed. All the remaining Percoll, except the dead cell and red blood cell pellet, was collected and washed, as intestinal macrophages do not accumulate within the leukocyte layer at the interface.

### Flow cytometry

Dead cells were stained using LIVE/DEAD (Thermo Fisher Scientific) for 15 min on ice in PBS. For traditional flow cytometry, nonspecific antibody binding was blocked by incubation with an anti-CD16/32 antibody (clone 2.4G2; University of California San Francisco antibody production facility) and whole mouse IgG (Sigma-Aldrich) on ice for 15 min, and the cells were stained with fluorophore-conjugated antibodies on ice for 20 min in PBS with 2 mM EDTA, 2% FCS, and 0.2% sodium azide. Cells were kept on ice and analyzed on a BD Fortessa (BD Biosciences). For spectral flow, cells were incubated with anti-CD16/32 APC-Fire750 and anti-CD64 PECy7 for 15 min on ice before the rest of the fluorophore-conjugated antibodies were added on ice for 20 min in Brilliant Stain Buffer (BD Biosciences). For GATA6 staining, cells were fixed and permeabilized with the eBioscience Foxp3/Transcription Factor Staining Buffer Set (Thermo Fisher Scientific) for 30 min, followed by staining with GATA6-PE (Cell Signaling Technologies) at 1:400 for 45 min on ice. Cells were kept on ice until analyzed on a 5 laser Cytek Aurora (Cytek Biosciences). Data were analyzed in FlowJo (FlowJo, LLC).

### Dimensionality reduction and unsupervised cluster identification

All analysis was performed within the FlowJo (v10.10) environment. For dimensionality reduction, equivalent numbers of cells from each sample were concatenated, and all markers were included except Live/Dead and GFP (for the RV transductants) using the UMAP R plug-in. The FlowSOM algorithm was applied to the concatenated cell populations for unsupervised cluster identification. These clusters and the characteristic marker expression that distinguish them within the UMAP were visualized using the ClusterExplorer plug-in or Color Mapping.

### Radiation bone marrow chimera generation

Recipient CD45.1 × CD45.2 F1 mice were lethally irradiated with a split dose of 500 rads in the evening of day −1 and 450 rads on the morning of day 0 using an XRad 300 X-ray producing machine (Precision Xray). Femur, tibia, and iliac bones from *LysM*^*Cre/+*^*Klf2*^*fl/fl*^, *LysM*^*+/+*^*Klf2*^*fl/fl*^, *LysM*^*Cre/+*^*Gata6*^*fl/fl*^, *LysM*^*+/+*^*Gata6*^*fl/fl*^, *LysM*^*Cre/+*^*Klf4*^*fl/fl*^, *LysM*^*+/+*^*Klf4*^*fl/fl*^, and *B6.SJL* mice were harvested, flushed with RPMI, and red blood cells were lysed with ACK buffer. Cells were counted and mixed 1:1 in appropriate combinations for the experiment. 6–8 million cells were injected i.v. into the tail vein of recipient mice in 100 µl of RPMI (Gibco). Mice were analyzed 8–12 wk after transfer.

### BMM culture and transduction

On day −1, 2.5 × 10^6^ 293T-GP2 RV producer cells were plated in 10 ml DMEM with 10 mM HEPES (Gibco), 1 mM sodium pyruvate (Gibco), 2 mM Glutamax (Gibco), 100 U/100 µg/ml penicillin/streptomycin (Gibco), 1.7 µl β-mercaptoethanol (Fisher), and 10% FCS (Gibco or Omega) (DM10+) in 10-cm TC-treated plates. On day 0, 293T-GP2s (RRID:CVCL_WI48; Clontech/Takara) were transfected with 100 ng MSCV plasmid (MSCV multiple cloning site IRES puroR 2A mCherry or GFP) and 10 ng pVSVg plasmid (8454; Addgene) with 3 µl/ml Lipofectamine 2000 (Thermo Fisher Scientific). Also on day 0, femur, tibia, and iliac bones from C57BL/6 mice were flushed with RPMI (Gibco), and red blood cells were lysed with ACK buffer (Gibco). Cells were counted, and 4 × 10^6^ cells were plated in 10-cm non-TC-treated plates in 8 ml RPMI with 10 mM HEPES (Gibco), 1 mM sodium pyruvate (Gibco), 2 mM Glutamax (Gibco), 100 U/100 µg/ml penicillin/streptomycin (Gibco), 1.7 µl β-mercaptoethanol (Fisher Scientific), 10% FCS (Gibco or Omega) (RP10^+^), and 10% titrated M-CSF 3T3 cell-conditioned laboratory-made media. On day 1, media was replaced on the 293T-GP2 with 8 ml. On day 2, viral supernatants were harvested from 293T-GP2s and filtered through 0.45-µM PES syringe filter. Bone marrow cultures were harvested, resuspended in 293T-GP2 supernatant supplemented with 10% M-CSF 3T3 cell media, and returned to the same 10-cm plate. Media was replaced on 293T-GP2s for repeating viral infections on day 3. On day 4, bone marrow cells are harvested out of plates and resuspended in 8 ml RP10^+^ with 10% M-CSF 3T3 supernatant with 5 µg/ml puromycin. Media was again replaced on day 7 with fresh RP10^+^ with 10% M-CSF 3T3 supernatant. For concurrent treatment with ATRA (Sigma-Aldrich), cells were given 1 µM ATRA daily starting on day 3. Cells were harvested on days 8–10 by incubating in 5 ml of 4 mM EDTA in PBS for 20 min in a 37°C incubator. EDTA was quenched by the addition of 7 ml RP10^+^. Cells were counted, and 1 × 10^5^ cells were resuspended in TRIzol (Thermo Fisher Scientific) and stored at −80°C for subsequent RNA isolation. The rest of the cells were analyzed by flow cytometry.

### BMM culture and transfer

On day −7, femur, tibia, and iliac bones from *LysM*^*Cre/+*^*Klf2*^*fl/fl*^ and *LysM*^*+/+*^*Klf2*^*fl/fl*^ mice were flushed with RPMI (Gibco), and red blood cells were lysed with ACK buffer (Gibco). Cells were counted, and 1 M cells were plated in 10-cm non-TC-treated plates in 8 ml RPMI with 10 mM HEPES (Gibco), 1 mM sodium pyruvate (Gibco), 2 mM Glutamax (Gibco), 100 U/100 µg/ml penicillin/streptomycin (Gibco), 1.7 µl β-mercaptoethanol (Fisher Scientific), 10% FCS (Gibco or Omega) (RP10^+^), and 10% titrated M-CSF 3T3 cell-conditioned laboratory-made media. On day −3, BMMs were given an additional 8 ml RP10^+^ with 10% M-CSF. On day −1, B6.SJL recipient mice were anesthetized with 90 mg/kg ketamine + 5 mg/kg xyalzine i.p. Upon achieving the appropriate anesthetic level, 5 ml of warm PBS was injected slowly into the peritoneal cavity with a 23-gauge needle. Mice were gently rocked, and then PBS was recovered with a 21-gauge needle. Mice were kept warm during the duration of the anesthesia and monitored until recovered. On day 0, BMMs were harvested by incubating in 5 ml of 4 mM EDTA in PBS for 20 min in a 37°C incubator. EDTA was quenched by the addition of 7 ml RP10^+^. Cells were counted and resuspended to inject 1 × 10^6^ cells per recipient mouse in 100 µl of RPMI i.p. Cells were harvested by lavage on day 7, stained, and sorted on live, single CD45^+^ cells with a BD FACS Aria Fusion (BD Biosciences) or analyzed by flow cytometry on a five-laser Cytek Aurora (Cytek Biosciences). TRIzol LS (Thermo) was added to sorted cells and frozen at −80°C for RNA extraction and library generation for RNA-Seq.

### Bone marrow monocyte purification and transfer

On day −1, JaxBoy recipient mice were anesthetized by i.p. administration of 90 mg/kg ketamine + 5 mg/kg xyalzine, and 5 ml of warm PBS was injected slowly into the peritoneal cavity with a 23-gauge needle. Mice were gently rocked and then PBS was recovered with a 21-gauge needle. On day 0, femur, tibia, and iliac bones from *LysM*^*Cre/+*^*Klf2*^*fl/fl*^ and *LysM*^*+/+*^*Klf2*^*fl/fl*^ mice were flushed with RPMI (Gibco). Cells were counted and monocytes were purified with the EasySep Mouse Monocyte Isolation Kit (STEMCell Technologies) according to the manufacturer’s instructions. 300,000 monocytes were checked for purity by flow cytometry (all samples >95% pure) before injection into the precleared JaxBoys i.p. Total peritoneal lavage was harvested 7 days later in 5 ml PBS + 2% FCS + 2 mM EDTA. Flow cytometry was performed with a five-laser Cytek Aurora analyzer (Cytek Biosciences).

### RNA-Seq library preparation, data mapping, and analysis of DEGs

Total RNA was isolated from cells using TRIzol or TRIzol LS reagent (Invitrogen). Chloroform extraction was performed according to the manufacturer’s protocol. RNA was recovered from the aqueous layer using RNA Clean and Concentrator-5 kit (Zymo Research). RNA was quantified with a Qubit (Invitrogen), and the integrity was evaluated with Agilent Bioanalyzer 2100 (Agilent Technologies). Libraries were prepared using the SMART-seq2 protocol (Picelli et al., 2014). For BMM-overexpressing transcription factors, samples were generated from three independent BMM transductions, but RNA was harvested, libraries were generated, and sequencing was all performed concurrently. Samples were sequenced on an Illumina NovaSeq sequencer using paired-end 50-base pair reads and were aligned with Kallisto v0.43.0 (Bray et al., 2016), using the “−b 100 and −t 4” options to obtain transcript abundances in transcripts per million and estimated counts. The kallisto index used during transcript quantification was built from the *Mus musculus* transcriptome GRCm38 from Ensembl (https://ftp.ensembl.org/pub/release-90/fasta/mus_musculus/cdna/). Transcript abundances were summarized to gene level using tximport (Soneson et al., 2015), and transcripts were annotated using the Bioconductor package biomaRt v3.18. Differences in gene expression between conditions were calculated, with correction for multiple comparisons, using DEseq2 in R v4.3.2 (Love et al., 2014). Genes with a log_2_ fold change ≥1 and Benjamini–Hochberg-adjusted P value ≤0.1 were termed significant DEGs.

We generated gene sets from the significant DEGs from our own previously sorted WT LCMs as compared with WT SCMs analyzed the same as above. We then created a custom gmx file containing one gene set with the 643 LCM-enriched genes and one gene set with the 381 SCM-enriched genes. We then compared the expression dataset we generated with the 13,801 genes expressed between the two transferred BMM populations (zero cross 7,717) or the 13,245 genes expressed between KLF2 OE BMMs to the EV control (zero cross 6,623) ranked by log_2_ fold change with 1,000 permutations in the classic enrichment score mode using the desktop GSEA software v4.3.3.

We used ggplot2 v3.5.0, ggrepel v0.9.4, ggfortify v0.4.16, and plotly v4.10.3 packages in R v4.3.2 to create scatterplots and perform linear regression modeling of shared DEGs from the comparison of LCMs to SCMs and the DEGs of transferred *LysM*^+/+^*Klf2*^fl/fl^ BMMs to *LysM*^Cre/+^*Klf2*^fl/fl^ BMMs or the DEGs of EV OE and KLF2 OE BMMs.

Volcano plots were generated in R with the EnhancedVolcano package v1.18.0. GO term enrichment for the AM data was performed with the DAVID Knowledgebase (v2023q3) web service.

### CUT&RUN and library prep

CUT&RUN was performed as previously described in Skene and Henikoff (2017) with modifications. Briefly, 600,000–800,000 LCM were sorted, and each sample was split in half. Cells were then washed and bound to magnetic Con A beads (Bangs Laboratories) in 200-µl PCR strip tubes. Cells were permeabilized with wash buffer containing 0.05% wt/vol digitonin (Sigma-Aldrich). Each sample was incubated at 4°C with anti-GFP (clone 290; Abcam) or rabbit IgG (Sigma-Aldrich) at a concentration of 50 μg/ml and left to rock overnight. Permeabilized cells were washed and incubated rotating at room temperature for 10 min with pA-MNase (kindly provided by the Henikoff lab, Fred Hutchinson Cancer Center, Seattle, WA, USA) at a concentration of 700 ng/ml. After washing, cells were incubated at 0°C, and MNase digestion was initiated by addition of CaCl_2_ to 1.3 mM. After 2 h, the reaction was stopped by the addition of EDTA and EGTA. Chromatin fragments were released by incubation at 37°C for 10 min. DNA was purified using DNA Clean & Concentrator-5 kit (Zymo). Libraries were prepared using the SMART-seq2 protocol (Picelli et al., 2014), quantified by Qubit (Thermo Fisher Scientific), and quality checked by Bioanalyzer (Agilent) before sequencing on an Illumina NovaSeq as paired ends to a depth of 3–5 million.

### CUT&RUN mapping, peak identification, and motif analysis

Adapter sequence and low-quality bases were trimmed using TrimGalore (v0.6.7) and then mapped to the mouse reference genome (GRCm39) using Bowtie2 (v2.4.2). Peak analysis was performed using SEACR (v1.3) with corresponding merged control samples. The number of reads mapped to spike-in *E. coli* was used to normalize each sample, and the top 1% of peaks are reported. To identify the motifs enriched in peak regions over the background, HOMER’s motif analysis (findMotifs.pl), including known default motifs and de novo motifs, was used (Heinz et al., 2010). IgG control peaks were used as background.

### Statistical analysis

Data were analyzed with Prism software or algorithm (GraphPad Software). Statistical details and group sizes are indicated in the figure legends.

### Online supplemental material

Fig. S1 shows the flow cytometry gating strategy for LCMs and SCMs, analysis of pleural cavity cells, quantification of monocytes and neutrophils, and unsupervised cluster identification and marker expression. Fig. S2 shows the flow cytometry gating strategy for macrophages in the liver, lung, small intestine, spleen, and transferred BMMs or monocytes, and phenotyping and quantification of *LysM*^*+/+*^*Klf2*^*fl/fl*^ or *LysM*^*Cre/+*^*Klf2*^*fl/fl*^ monocytes transferred in the peritoneal cavity. Fig. S3 shows the GFP expression in LCMs from GFP-KLF2 knock-in-expressing mice and CUT&RUN peaks for additional LCM markers. Fig. S4 shows the scatter plot of DEGs from LCMs versus SCMs compared with DEGs from KLF2 OE BMMs versus EV. Fig. S5 shows the phenotyping and quantification of cells from the BAL of KLF4-deficient mice, RNA-Seq results comparing *LysM*^*+/+*^*Klf4*^*fl/fl*^ AMs to *LysM*^*Cre/+*^*Klf4*^*fl/fl*^ AMs, and GO term analysis of DEGs from that RNA-Seq analysis. Table S1 shows the DEGs of LCMs and SCMs. Table S2 shows the DESeq2 results for transferred *LysM*^*+/+*^*Klf2*^*fl/fl*^ versus *LysM*^*Cre/+*^*Klf2*^*fl/fl*^ BMMs on day 7. Table S3 shows the DESeq2 results for KLF2OE BMMs relative to EV. Table S4 the shows DESeq2 results for GATA6OE BMMs relative to EV. Table S5 shows the DESeq2 results for sorted *LysM*^*+/+*^*Klf4*^*fl/fl*^ versus *LysM*^*Cre/+*^*Klf4*^*fl/fl*^ AMs. Table S6 shows the antibodies used for flow cytometry.

## Supplementary Material

Table S1 shows the DEGs of LCMs and SCMs.

Table S2 shows the DESeq2 results for transferred *LysM*^+/+^*Klf2*^fl/fl^ versus *LysM*^Cre/+^*Klf2*^fl/fl^ BMMs on day 7.

Table S3 shows the DESeq2 results for KLF2OE BMMs relative to EV.

Table S4 shows the DESeq2 results for GATA6OE BMMs relative to EV.

Table S5 shows the DESeq2 results for sorted *LysM*^+/+^*Klf4*^fl/fl^ versus *LysM*^Cre/+^*Klf4*^fl/fl^ AMs.

Table S6 shows the antibodies used for flow cytometry.

## Data Availability

Sequencing results are available from the Gene Expression Omnibus public database (GSE288281 and GSE288282). All other data are available in the main text or the supplementary materials. All materials (cell lines, mice, and plasmids) are available upon request and after completion of a material transfer request to the corresponding author (G.M. Barton).
